# Electrical Source Imaging in Freely Moving Rats: Evaluation of a 12-Electrode Cortical Electroencephalography System

**DOI:** 10.3389/fninf.2020.589228

**Published:** 2021-01-25

**Authors:** Stanislav Jiricek, Vlastimil Koudelka, Jaroslav Lacik, Cestmir Vejmola, David Kuratko, Daniel K. Wójcik, Zbynek Raida, Jaroslav Hlinka, Tomas Palenicek

**Affiliations:** ^1^National Institute of Mental Health, Klecany, Czechia; ^2^Department of Cybernetics, Faculty of Electrical Engineering, Czech Technical University in Prague, Prague, Czechia; ^3^Department of Complex Systems, Institute of Computer Science of the Czech Academy of Sciences, Prague, Czechia; ^4^Department of Radioengineering, Faculty of Electrical Engineering and Communication, Brno University of Technology, Brno, Czechia; ^5^Third Faculty of Medicine, Charles University, Prague, Czechia; ^6^Laboratory of Neuroinformatics, Nencki Institute of Experimental Biology of the Polish Academy of Sciences, Warsaw, Poland

**Keywords:** electroencephalography, preclinical models, electrical source imaging, translational research, auditory steady-state response experiment, fieldtrip

## Abstract

This work presents and evaluates a 12-electrode intracranial electroencephalography system developed at the National Institute of Mental Health (Klecany, Czech Republic) in terms of an electrical source imaging (ESI) technique in rats. The electrode system was originally designed for translational research purposes. This study demonstrates that it is also possible to use this well-established system for ESI, and estimates its precision, accuracy, and limitations. Furthermore, this paper sets a methodological basis for future implants. Source localization quality is evaluated using three approaches based on surrogate data, physical phantom measurements, and *in vivo* experiments. The forward model for source localization is obtained from the FieldTrip-SimBio pipeline using the finite-element method. Rat brain tissue extracted from a magnetic resonance imaging template is approximated by a single-compartment homogeneous tetrahedral head model. Four inverse solvers were tested: standardized low-resolution brain electromagnetic tomography, exact low-resolution brain electromagnetic tomography (eLORETA), linear constrained minimum variance (LCMV), and dynamic imaging of coherent sources. Based on surrogate data, this paper evaluates the accuracy and precision of all solvers within the brain volume using error distance and reliability maps. The mean error distance over the whole brain was found to be the lowest in the eLORETA solution through signal to noise ratios (SNRs) (0.2 mm for 25 dB SNR). The LCMV outperformed eLORETA under higher SNR conditions, and exhibiting higher spatial precision. Both of these inverse solvers provided accurate results in a phantom experiment (1.6 mm mean error distance across shallow and 2.6 mm across subcortical testing dipoles). Utilizing the developed technique in freely moving rats, an auditory steady-state response experiment provided results in line with previously reported findings. The obtained results support the idea of utilizing a 12-electrode system for ESI and using it as a solid basis for the development of future ESI dedicated implants.

## 1. Introduction

In neuroscience, the rat model is widely used for studying brain disorders and investigating brain functions (Ellenbroek and Youn, 2016). The relatively large size of the rat facilitates various types of surgical procedures (Kjell and Olson, 2016) including neurosurgical manipulations such as implantation of electrodes. Despite the fact that functional neuroimaging methods are now a gold standard in studying brain functions in humans, the same approaches are still relatively anecdotal in rodent models. Nevertheless, a variety of these methods including functional magnetic resonance imaging (fMRI) (Febo, 2011; Bartelle et al., 2016), positron emission tomography (Zimmer et al., 2014), electroencephalography (EEG) (Drinkenburg et al., 2015), diffuse optical tomography (DOT) (Kim et al., 2014), and functional ultrasound (Macé et al., 2011) have already been used in rodents. While neuroimaging methods monitor brain activity as accurately as possible, one of the limitations of using these in rodents is the relatively small spatial resolution due to the small size of the brain.

EEG in contrast to fMRI or PET has a relatively low spatial resolution, but a much higher temporal resolution enabling monitoring events within milliseconds. Therefore, the high temporal resolution makes this method suitable for monitoring fast components of sensorimotor as well as cognitive processes also in rodents. However, due to the small volume of the rat brain (approximately 2 cm^3^), the volume conduction effect and low skull conductivity (Welniak-Kaminska et al., 2019), spatially accurate electrical source imaging (ESI) for testing small deep sources in the rat brain has not been extensively explored yet and remains a challenging and difficult task.

Several invasive, non-invasive, and combined rodent EEG electrode systems, mostly for translational research, have been introduced over the last decade. These systems differ in the number of electrodes, their positions, and the consequent need for surgery. One direction deals with a higher density of electrodes in order to improve spatial resolution. For example, a polyimide-based micro-electrode array consisting of 32 electrodes and placed directly on the skull has been used for high-resolution brain mapping of seizures in freely moving mice (Choi et al., 2010; Lee et al., 2013), or a combined system of 32 electrodes regularly placed on the skull, and 16-channel intracortical recording has been proposed and tested in somatosensory evoked potential (SEP) brain mapping in anesthetized mice (Mégevand et al., 2008; Quairiaux et al., 2011). Another approach combines EEG with recording manifestations of cellular metabolism equivalent to a human. Sumiyoshi et al. (2011) introduced a 31-electrode mini-cap for simultaneous EEG and fMRI SEP recording in rodents, while Franceschini et al. (2008) used six electrodes for EEG recording placed around a DOT sensor. On the contrary EEG recording telemetry systems introduced in several studies (White et al., 2006; Chang et al., 2011; Lundt et al., 2016) used only a few electrodes and are designed for long-term monitoring, mostly in epilepsy or circadian research. For detecting exact sources of epileptic seizures, a system combining skull and depth electrodes was proposed by Van Nieuwenhuyse et al. (2015). Finally, Steinmetz et al. (2020) have devised approaches using high density implantable probes such as Neuropixels for extremely precise localization of the sources within the surrounding brain tissue. In contrast to the above mentioned methods with a higher density of electrodes and ESI, due to spatial issues these approaches are limited by the number of areas that can be explored simultaneously.

In contrast to the thousands of studies using standard recordings of electrical activity from various types of electrode systems, only a few have attempted to use ESI in rodents. Bae et al. (2015) localized sources within the brain of an anesthetized Wistar rat using a non-invasive 32-channel EEG mini-cap introduced by Riera et al. (2012) with a forward model based on the finite-element method (FEM) and the inverse methods of low-resolution brain electromagnetic tomography and exact low-resolution brain electromagnetic tomography (eLORETA). Valdés-Hernández et al. (2019) validated this EEG mini-cap system in terms of optimal electrode separation using the half and fifth sensitivity volume method. ESI was also performed in anesthetized mice using non-invasive 40-channel micro-array EEG, and the accuracy of three inverse solutions—minimum norm estimates (MNE), multiple signal classification, and single dipole fitting—was compared using the boundary-element method based forward model (Lee et al., 2013). Integrating a DOT sensor and electrode array in rats, Yang et al. (2015) showed that prior information obtained from the DOT may improve the accuracy of the minimum norm based solution. In a previous study on physical phantom measurements (Yang and Jiang, 2013), a significant bias of ESI was observed.

The major issue of rodent studies is that most of them lack translational validity as there are no guidelines for animal EEG. While in humans, EEG is typically recorded during a resting state with eyes closed or open, and deals with standard electrode placement and recording conditions and analytical procedures, EEG studies in animals have many more variables, such as recording in anesthetized animals, contamination of EEG with behavioral activity, testing during the inverse phase of the cycle etc. Therefore, we have attempted to develop a method that would be applicable in a translational manner.

As our team has worked with rats for several years using 12- and 19- electrode systems developed at the National Institute of Mental Health (Klecany, Czech Republic) (Páleńıček et al., 2011, 2013), we had access to a significant amount of data for testing our method. These data were collected mainly during our pharmaco-EEG experiments and include baseline recordings (without any pharmacological manipulation) accompanied with records of behavioral activity and inactivity of the animals. In our previous work, we proposed that the segments of EEG that correspond to behavioral activity are more likely to be a model of resting EEG in humans, whereby having translational validity (Fujáková et al., 2014).

Therefore, the main aim of the present work is to investigate the precision of source localization within the Wistar rat brain for data collected with a 12-electrode system. To the best of our knowledge, this is the first paper to evaluate an ESI system in freely moving rats. In order to achieve this aim, a comprehensive evaluation procedure involving simulated data, physical phantom measurements, and *in vivo* experiments was performed. As recent studies mostly utilized mostly minimum norm based solutions to an inverse problem, this paper also applies a minimum variance based solution to compare these two approaches. As such, the present study lays a solid foundation for ESI systems in freely moving rats.

We believe that the development of a reliable tool for locating electrical sources in the rat's brain will enable analytical procedures to be performed analogous to those used in human EEG analysis and, whereby allowing the application of advanced methods of analysis such as functional connectivity, entropy, wavelet analysis, or graph theory in three-dimensional (3D) space. Using this approach in combination with deep electrodes also enables exact verification of the estimated sources and/or hubs of large scale networks. Last but not least, our approach enables the use of non-invasive or less-invasive methods of data collection, which are capable of reaching sources in subcortical areas while reducing suffering of animals and as such have a significant potential for the standardized evaluation of EEG data from preclinical models.

## 2. Materials and Equipment

**Figure 1:**
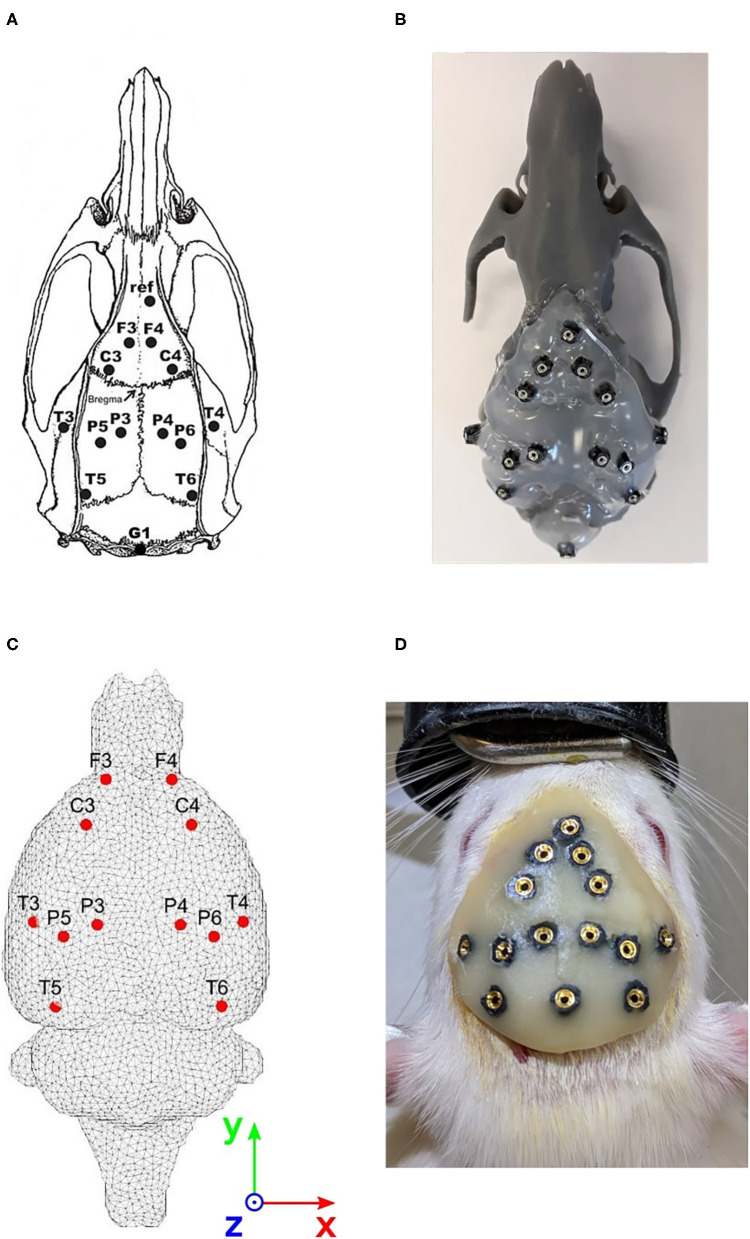
Approach for evaluating the cortical electrode system. **(A)** Electrode positions on the rat's skull. Coordinates refer to bregma. F3/ F4: frontal association cortex (A = +5.0 mm, L = ± 2.0 mm). C3/ C4: primary motor cortex (A = +2.2 mm, L = ± 3.2 mm). P3/P4: medial parietal association cortex (A = −3.8 mm, L = ± 2.5 mm). P5/P6: lateral parietal association cortex (A = −4.5 mm, L = ± 4.5 mm). T3/T4: secondary auditory cortex (A = −3.6 mm, L = ± 7.2 mm). T5/T6: temporal association cortex (A = −8.3 mm, L = ± 5.8 mm). Ref: reference electrode (above the olfactory bulb). A = Anteriorly (+) or posteriorly (−) from the bregma; L = laterally from the bregma. **(B)** 3D printed phantom of the rat's brain. **(C)** Head model with registered cortical electrode positions. **(D)** Implanted electrode system in the rat's skull.

A 12-electrode system developed for registering EEG signals in freely moving rats was used (Páleńıček et al., 2011, 2013). The electrode layout is shown in Figure 1A. The main features of the system may be summarized as follows:

- This is a cortical EEG system. The electrodes are implanted such that they are touching the surface of the cortex.
- The surgery is performed 7 days before the EEG recording.
- Electrode positions are chosen according to known functional areas in the rat brain, so the recordings may be compared with those obtained with standard human EEG systems.
- The typical value of electrode impedance is approximately 5 kΩ.

Data were acquired with a BioSDA09 standard 48-channel digital EEG amplifier (M&I Ltd., Prague, Czech Republic) with an application range of 0.01–400 Hz for sampling frequency 1 kHz (*in vivo* experiments) and 0.01–2,000 Hz for sampling frequency 5 kHz (phantom experiments).

For the testing of the phantom, an IQ SIGLENT SDG6022X generator was used. The fabricated phantom was scanned with a Siemens Somatom64 computed tomography (CT) scanner to precisely locate the source dipoles and sensing electrodes.

The stimuli presentation was implemented in Opensezame software (Mathôt et al., 2012). An AQ M4 (AQ, Czech Republic) audio amplifier was used for stimuli delivery. An Arduino-based device was designed to ensure precise onsets of the auditory stimuli.

## 3. Methods

**Figure 2:**
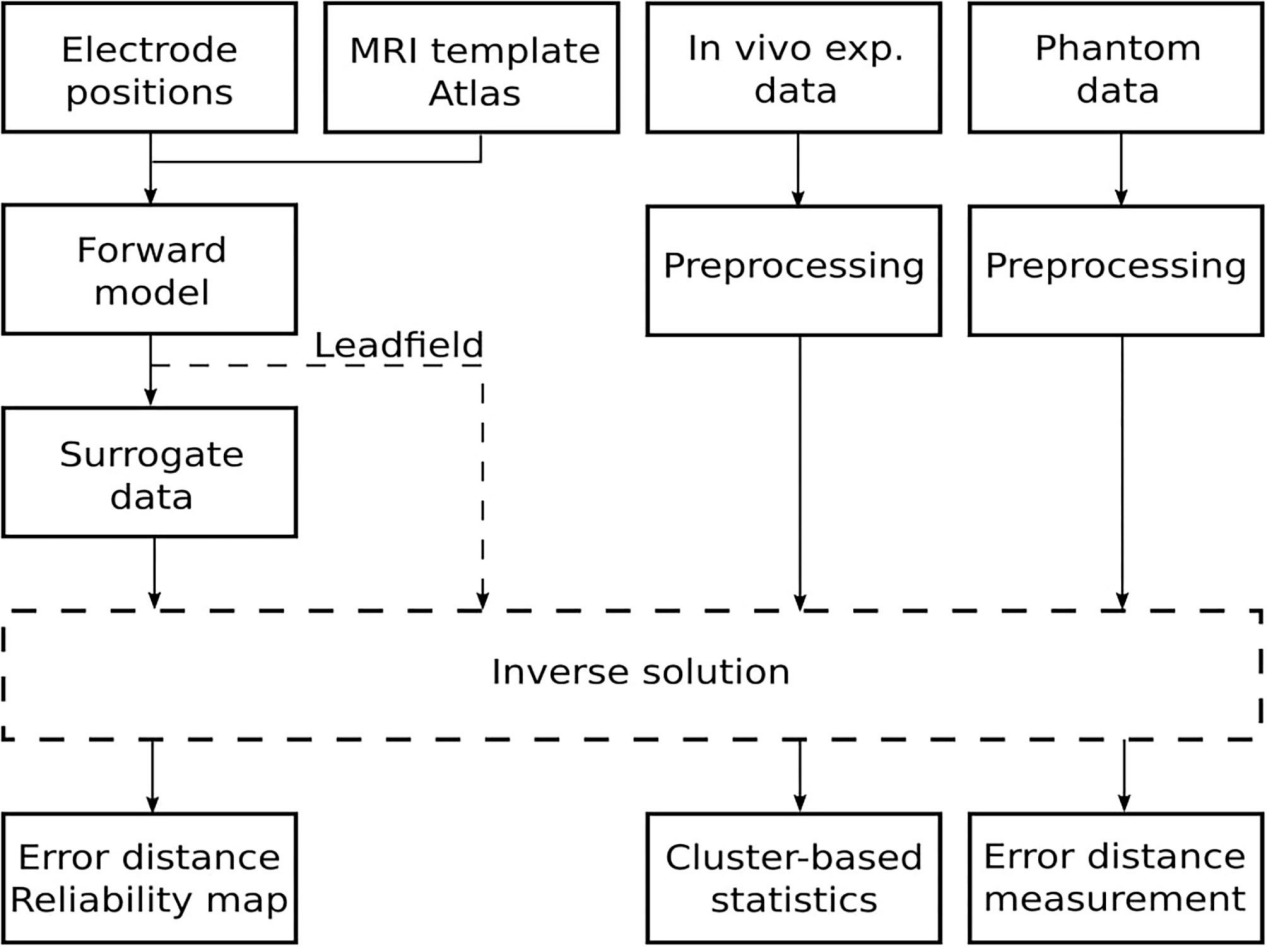
Schematic flowchart of the approach for evaluating the quality of source localization.

This section describes the methods for the 12-electrode system implantation, the auditory steady-state response (ASSR) experiment design, phantom fabrication, and the evaluation based on the approach depicted in Figure 2. Specifically, the forward model depicted in Figure 1C was based on a realistically shaped magnetic resonance imaging (MRI) scan of the rat's brain and use of the FEM to implement Poisson's equation, finally obtaining the lead field matrix (Hallez et al., 2007). Forward modeling is described in detail in section 3.1. Brain activity was estimated using four inverse solutions: standardized low-resolution brain electromagnetic tomography (sLORETA) (Pascual-Marqui, 2002), exact low-resolution brain electromagnetic tomography (eLORETA) (Pascual-Marqui, 2007), linear constrained minimum variance (LCMV) (Van Veen et al., 1997), and dynamic imaging of coherent sources (DICS) (Groß et al., 2001). The inverse solutions used are described in section 3.2. Based on simulated surrogate data, the phantom measurements, and the *in vivo* experimental data, it was possible to critically evaluate the capabilities of this cortical electrode system in terms of ESI. The precision of the inverse methods on the surrogate data generated by the lead field matrix is evaluated in section 3.3. The precision of the inverse methods on data obtained with the electrode system on the phantom and *in vivo* experiments is evaluated in sections 3.4–3.6, respectively. While surrogate data and phantom measurement approaches provide ground truth data against which the reconstruction may be gauged directly, evaluating the precision of a reconstruction for *in vivo* experimental data requires non-parametric statistical testing.

### 3.1. Forward Problem of EEG

To localize sources within the rat's brain from EEG recordings, it is necessary to first solve the so-called forward problem. ESI is a linear inverse problem and takes the form of a Fredholm integral equation of the first kind:

(1) $$\begin{array}{l} \int_{\Omega }K\left(s,v\right)y\left(v\right)dv=x\left(s\right), \end{array}$$

where the kernel *K*(*s, v*) and *x*(*s*) are known functions, while *y*(*v*) is to be estimated (Hansen, 2010). Here, *y*(*v*) represents the spatial distribution of brain activity in a region Ω, *x*(*s*) represents the potential measurement on the surface of the brain, and *K*(*s, v*) determines a linear relationship between *y* and *x* (Koudelka et al., 2018). A closed-form expression for *K*(*s, v*) in ESI is known for simplified models such as those in free space or in a sphere, whereas for realistic brain shapes and a finite number of electrodes, the problem needs to be discretized over space and time (Hallez et al., 2007):

(2) $$\begin{array}{l} X=KY+e, \end{array}$$

where *X* is a matrix of data measurements with dimensions *N* × *T* (number of electrodes × discrete time samples). Matrix *Y* with dimensions *P* × *T* (number of sources × discrete time samples) represents the current dipole moments that model the brain activity. Finally, matrix *K* with dimensions *N* × *P* is called the gain matrix or the lead field. The lead field is an electric current field in the volume conductor generated by feeding a unit current to the lead (Malmivuo et al., 1997). In other words, *K* is a weighting matrix where each element contains the electrical potential on the electrode *n* due to the unit activity of the current dipole moment *p*. The last term on the right-hand side of Equation (2) denotes additive noise in the measurements. The purpose of forward modeling is to obtain the *K* matrix with respect to certain prior assumptions of the problem.

This paper used the FieldTrip-SimBio pipeline for solving the forward problem (Oostenveld et al., 2011; Vorwerk et al., 2018). A volumetric dataset of the MRI scan of a rat's brain in the NIfTI format with approximately 6 × 10^7^ regular voxels of edge length 0.05 mm, and the corresponding rat brain atlas (Calabrese et al., 2013) indexing 27 anatomical areas was provided by the 3D Brain Atlas Reconstructor (Majka et al., 2012) (www.3dbar.org). Both datasets are based on the Waxholm space reference, which is advantageous for co-registration (Johnson et al., 2010). The volumetric MRI scan was down-sampled by a factor of 2, and a threshold segmentation was applied to obtain a mask defining the brain volume. A single-compartment tetrahedral mesh was generated, consisting of 38985 nodes and 231,326 tetrahedrons. Subsequently, a head model was created based on SimBio software (Vorwerk et al., 2018) implemented in the FieldTrip toolbox, which assigns a homogeneous isotropic electrical conductivity of 0.33 S/m to the whole volume. According to Hallez et al. (2007), the rat brain conductivity fits the conductivity in the human brain. Twelve active electrode positions of the cortical EEG system were co-registered with the brain volume and placed to the nearest node on the surface of the head model. Electrode positions on the head model are depicted in Figure 1C.

The last step before lead field computation was to define a reliable source model. For this purpose, current dipoles were placed at nodes of the regular volumetric grid with an edge length of 1 mm. Based on the atlas used from Majka et al. (2012), 27 anatomical areas were assigned to the source dipole positions. Selected deep functional structures were included in the source model additional to the isocortex area. The criterion for including or excluding brain areas was based on areas expected to contribute to the EEG signal measured by the 12-electrode system on the brain surface, see Table 1. Based on this assumption, only current dipoles belonging to one of the electrically active areas were preserved. Source model positions are visualized on the MRI image in the figure included in the Supplementary Material. It is evident that restricting the source model results in better accuracy of ESI. Due to the relatively large volume one dipole represents, dipole orientations as another source model restriction were not assumed. The St. Venant approach approximated dipoles in the discrete head model (Medani et al., 2012). Finally, the lead field matrix for 1,557 dipoles was computed.

**Table 1:** Twenty-seven anatomical areas of the rat brain based on an atlas (Majka et al., 2012).

**Source model assumptions**	
**Included**	**Excluded**
Accumbens	Brainstem
Amygdala	Diagonal domain
Bed n. stria terminalis	Hindbrain
Cerebellum	Pituitary
Isocortex	Olfactory structures
Pallidum	Pineal gland
Hippocampal formation	Anterior commissure
Hypothalamus	Corpus callosum
Diencephalon	Cingulum
Midbrain	Fimbria fornix
Preoptic area	Internal capsule
Substantia nigra	Optic pathway
Septum	Ventricles
Striatum	

*Areas are divided according to prior assumptions on their electrical activity registered by the electrode system*.

### 3.2. Inverse Problem of EEG

A variety of inverse solutions exist for estimating current source parameters of the distributed source model. The goal is to find the inverse operator such that after multiplying it with the measured data, it gives rise to an estimate of the source activity that is as reliable as possible. Some inverse solutions are defined as regularized least-squares problems by Tikhonov regularization, such as MNEs and their generalizations. Other inverse solutions are based on spatial filtering and are called beamformers (Grech et al., 2008). Despite the known limitation of beamforming techniques that they cannot localize spatially separate but temporally correlated sources, they are widely used in neuroscience. A one minimum norm based eLORETA solution and two spatial filtering inverse solutions were used for estimating the source activity sLORETA, LCMV, and its version in a frequency domain DICS. There are two families of spatial filters commonly applied: minimum variance and minimum norm (Jonmohamadi et al., 2014). LCMV represents the minimum variance spatial filter, whereas sLORETA represents the minimum norm one. The principle of spatial filtering is to design the spatial filter *w*^*T*^ that allows the signal to pass through for a certain source *p* and suppresses the signal from all other source locations. This approach may be expressed as follows:

(3) $$\begin{array}{l} y_{p}=w_{p}^{T}X, \end{array}$$

where *y*_*p*_ is a dipole moment of the source *p* with typical dimensions 3 × *T* (three dipole orientations × discrete time samples), $w_{p}^{T}$ denotes a spatial filter for the source *p*, and *X* is the matrix of the measured data. Mathematically, the LCMV beamformer problem may be expressed as (Van Veen et al., 1997):

(4) $$\begin{array}{l} \underset{w_{p}}{\min}w_{p}^{T}C_{X}w_{p}, \end{array}$$

subject to $w_{p}^{T}K_{p}=1$. *C*_*X*_ denotes a covariance matrix of the measured data, and *K*_*p*_ is the lead field of the *p*-th dipole. The solution to Equation (4) using the method of Lagrange multipliers is:

(5) $$\begin{array}{l} w_{p}^{T}={\left(K_{p}^{T}C_{X}^{-1}K_{p}\right)}^{-1}K_{p}^{T}C_{X}^{-1}. \end{array}$$

Combining Equations (3) and (5), the variance Var_*p*_ of the dipole moment *p* that represents the source activity may be obtained:

(6) $$\begin{array}{l} \text{Va}{\text{r}}_{p}=\text{Tr}\left[{\left(K_{p}^{T}C_{X}^{-1}K_{p}\right)}^{-1}\right], \end{array}$$

where Tr denotes the trace of a matrix. Note that the variance is location dependent because the covariance matrix is multiplied by the lead field matrix for a given source. As deep sources have small elements in the lead field matrix, the inverse expression in Equation (6) becomes high and vice versa with sources close to the electrodes. Therefore, to obtain a spatial map of the neural activity, the variance is noise normalized, and the expression is called a neural activity index (NAI):

(7) $$\begin{array}{l} \text{NA}{\text{I}}_{p}=\frac{\text{Tr}\left[{\left(K_{p}^{T}C_{X}^{-1}K_{p}\right)}^{-1}\right]}{\text{Tr}\left[{\left(K_{p}^{T}Q^{-1}K_{p}\right)}^{-1}\right]}, \end{array}$$

where *Q*^−1^ is a noise covariance matrix. For simplicity, it is substituted by the identity matrix. To decrease sensitivity to noise, regularization is involved. A regularized LCMV solution is achieved using ${\left(C_{X}^{-1}+\gamma I\right)}^{-1}$ instead of $\left(C_{X}^{-1}\right)$, where γ is the regularization parameter; in this work, it is estimated by γ = 0.003λ, where λ is the highest eigenvalue of the covariance matrix $C_{X}^{-1}$ (Sekihara et al., 2001). In the DICS case, the solution is obtained in the same sense as LCMV, see Equation (6), but the cross-spectral density matrix of a specific frequency replaces the covariance matrix. Hence, this solver is beneficial when estimating sources that show activity in a certain frequency band.

The sLORETA minimum norm beamformer problem takes the form:

(8) $$\begin{array}{l} \underset{w_{p}}{\min}w_{p}^{T}K_{p}K_{p}^{T}w_{p}, \end{array}$$

subject to $w_{p}^{T}K_{p}K_{p}^{T}w_{p}=1$. This approach uses an estimate provided by the MNE inverse operator: $K_{p}^{T}{\left(K_{p}K_{p}^{T}+\gamma I\right)}^{-1}$. This estimate is then standardized by its variance Var_*Y*_, which has the form of the resolution matrix: $K_{p}^{T}{\left(K_{p}K_{p}^{T}+\gamma I\right)}^{-1}K_{p}$. Finally, the estimated source density of the sLORETA solution for dipole *p* is:

(9) $$\begin{array}{l} Y_{p_{\text{sLOR}}}=Y_{p_{\text{NAI}}}^{T}{\left[\text{Va}{\text{r}}_{Y}\right]}_{pp}^{-1}Y_{p_{\text{NAI}}}, \end{array}$$

where *Y*_*p*_NAI__ is the inverse solution obtained by the MNE inverse operator and [Va_r_*Y*_]*pp*_ is the *p*-th 3 × 3 diagonal block of the matrix Var_*Y*_. While MNE is known for its poor estimate of deep sources, according to Grech et al. (2008), sLORETA has zero location error when estimating single sources.

The eLORETA algorithm is derived from a weighted minimum norm problem, the solution of which can be expressed as follows (Pascual-Marqui et al., 2011):

(10) $$\begin{array}{l} Y_{p_{\text{eLOR}}}={W_{p}}^{-1}{K_{p}}^{T}{\left(K_{p}{W_{p}}^{-1}{K_{p}}^{T}+\gamma I\right)}^{-1}X, \end{array}$$

where *W* denotes the weighting matrix preserving zero localization error over the volume in terms of the accuracy under the zero-noise condition. The weighting matrix satisfying such a criterion has to be found numerically by solving the following equation:

(11) $$\begin{array}{l} W_{p}={\left[{K_{p}}^{T}{\left(KW^{-1}K^{T}+\gamma I\right)}^{-1}K_{p}\right]}^{-1/2}. \end{array}$$

Regularization parameters for LCMV and DICS beamformers are computed from the highest eigenvalue of the covariance and cross-spectral matrices, respectively, see Jonmohamadi et al. (2014). Regularization parameters for eLORETA and sLORETA are computed utilizing a leave-one-out cross-validation method.

### 3.3. Evaluation on Surrogate Data

The three above-mentioned and previously described regularized inverse solutions sLORETA, eLORETA, and LCMV were compared by estimating single dipole simulations according to the following scenario. In each of the source model dipole positions, a dipole moment time course of a 0.1 s duration was generated as white Gaussian noise with a sampling frequency of 1,000 Hz, i.e., 100 time samples in total. The number of time samples was chosen as a trade-off between a sufficient amount of data for computing the covariance matrix while not disadvantaging the sLORETA and eLORETA solvers, which do not use the covariance matrix for calculating filters. Potential on the electrodes was computed by multiplying the dipole moment time course by the corresponding lead field matrix. Due to unconstrained dipole moment directions, the potential and following steps were performed separately for the *x*, *y*, and *z* directions of the Cartesian coordinate system. According to Equation (2), white Gaussian noise representing additive noise of the measurement was added uniformly to the electrode potential time courses under four levels of signal to noise ratio (SNR) conditions: 5, 10, 15, and 25 dB using the Matlab built-in *awgn* function. For each dipole position, each dipole moment direction and each level of SNR, sLORETA, eLORETA, and LCMV inverse solutions were performed. While the regularization parameter for LCMV was obtained from the highest eigenvalue of the covariance matrix, the regularization parameter for eLORETA was determined as a mean value obtained from leave-one-out cross-validation over all dipole positions for each of the SNR values. Based on the findings of this study, the sLORETA solution was not sensitive to the value of the regularization parameter obtained from leave-one-out cross-validation in this model. Therefore, the default value of the regularization parameter 5% of the mean value on the diagonal of the data covariance matrix was used. Source localization quality was evaluated by error measurements (Grech et al., 2008) and reliability maps (Chintaluri et al., 2019). Two error distance criteria ED1 and ED2 were calculated for every position of the simulated dipole. The ED1 criterion is defined as the Euclidean distance between the simulated dipole position and the position of the maximum estimated by inverse solutions:

(12) $$\begin{array}{l} \text{ED}1\left(p\right)=|r_{p_{\max}}-r_{p}|, \end{array}$$

where *r*_*p*_max__ is the position of the estimated maximum and *r*_*p*_ is the position of the simulated dipole activity. Whereas the ED1 criterion involves only the bias of the global maximum, the ED2 criterion also includes other activation defined as local maxima. Error distances of all local maxima are weighted as a proportion of their activation value relative to the activation of the global maximum and summed as follows:

(13) $$\begin{array}{l} \text{ED}2(p)=\sum_{l=1}^{L}\left|r_{p_{l}}-r_{p}\right|.\;\left|\frac{Y_{p_{l}}}{Y_{p_{\max}}}\right|, \end{array}$$

where *r*_*p*_*l*__ is the position of the local maximum *l* for a dipole position *p*, *Y*_*p*_*l*__ is its activation value, and *Y*_*p*_max__ is the activation value of the global maximum. In general, these two error distance criteria give an insight into how biased the estimated solution will be, if activation is expected in a particular part of the brain. However, in most cases, researchers will ask to what extent they can trust the activation in a particular part of the brain if they do not assume activation in any specific location. To try to at least partially answer this question, reliability maps may be utilized (Chintaluri et al., 2019). For such a calculation, a family of brain activations needs to be defined. In continuous brain models, a family of dipoles is defined by a variety of functions; in this discrete model, the family of activation is defined in the same manner as in the previous error distance criteria, i.e., by single dipole activations in each location of the source model by white Gaussian noise time courses in three fixed perpendicular orientations separately. A reliability map (Chintaluri et al., 2019) is then defined as:

(14) $$\begin{array}{l} \text{Reliability}(p)=\;\frac{\sum_{i=1}^{I}\left|\frac{Y_{p_{i,\text{est}}}}{\left\|Y_{i_{\text{est}}}\right\|}-\frac{Y_{p_{i,\text{sim}}}}{\left\|Y_{i_{\text{sim}}}\right\|}\right|}{I}, \end{array}$$

where *Y*_*p*_*i*,est__ denotes the estimated activity at the *p* source model position and the *i*-th test simulation, ∥*Y*_*i*_est__∥ is the norm of the estimated activity in the *i*-th simulation, *Y*_*p*_*i*,sim__ is the simulated activity on the *p* source model position and the *i*-th test simulation, and ∥*Y*_*i*_sim__∥ is the norm of the simulated activity in the *i*-th simulation. The obtained maps calculated for each solver and each SNR level serve for critical evaluation of the model and possible subsequent modification of parameters in each step of the source localization.

### 3.4. Evaluation on Phantom Measurements

**Figure 3:**
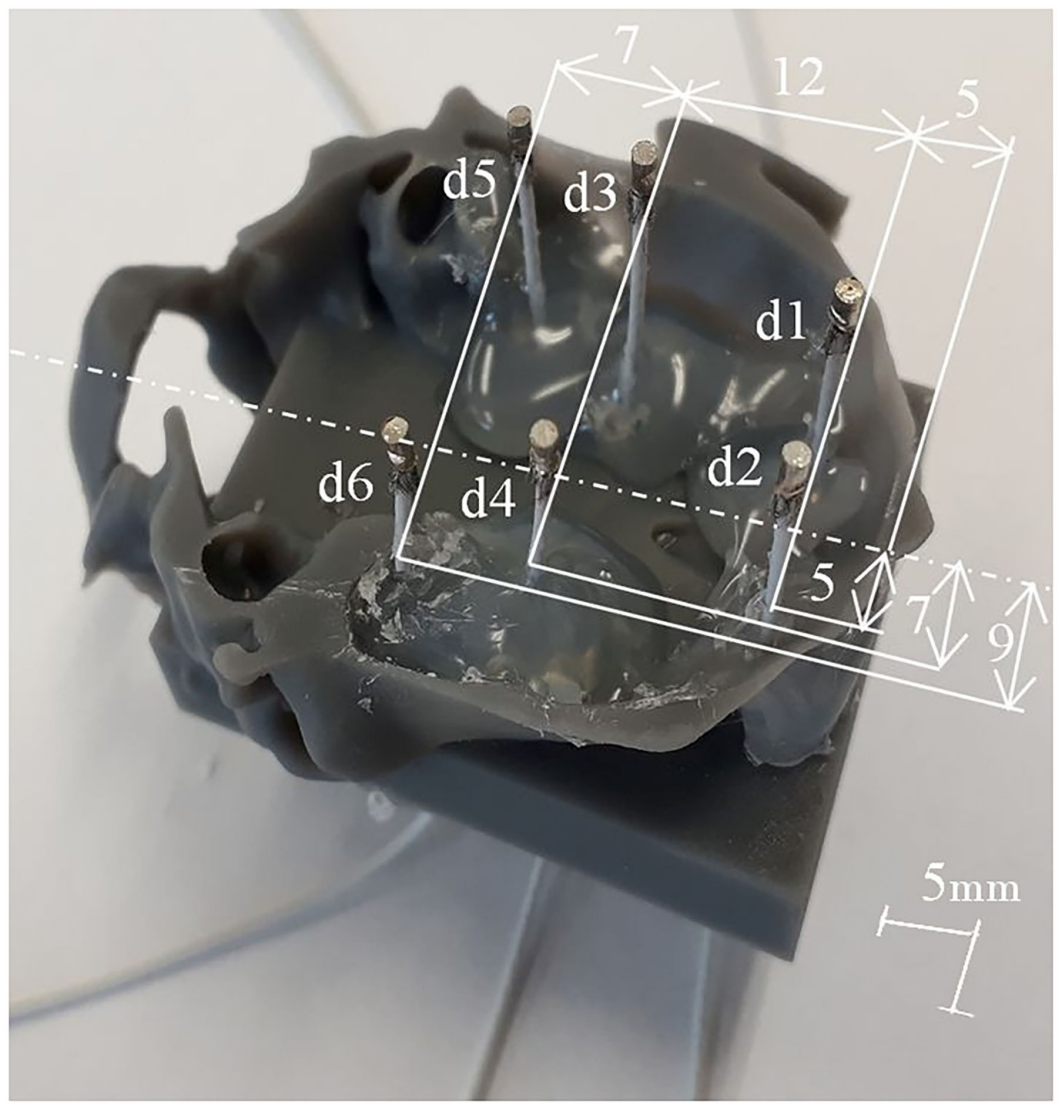
Fabrication of six physical excitation dipoles for a shallow phantom. Deep excitation dipoles were designed and fabricated in the same way.

To evaluate inverse solutions, a realistic homogeneous phantom of the rat's head was created (Lacik et al., 2020). It consisted of the following main parts: skull, brain, excitation dipoles, and sensing electrodes. The skull was based on the realistic skull of a 5-month-old female whose digital model was made using CT. The obtained model was further slightly modified. Small holes were removed, a low-profile brick was added to have a stable support for the excitation dipoles, and it was split into two parts. Both parts were scaled by 1.8 (the scaling is allowed by a linear operating regime of the phantom, Lacik et al., 2020) and printed. For printing, a 3D printer based on stereolithography technology and a Formlabs standard resin (Formlabs, USA) was used. The brain was modeled by a homogeneous medium based on a mixture of deionized water, sodium chloride (NaCl), and agar. Since most of the neural activity is concentrated in the gray matter, the mixture was modified to have a conductivity of 0.33 S/m. Six excitation dipoles were fabricated using a thin coaxial cable with a total length of 4.5 mm, to model neural activity of the brain. To monitor the surface electric potential, small pins were used as sensing electrodes. The outer electrically conductive surfaces of the dipoles and electrodes were platinum plated. To complete the phantom, 14 sensing electrodes and excitation electric dipoles were fixed on the upper (Figure 1B) and the lower part (Figure 3) of the skull using a hot melted glue gun, respectively. Then, both parts of the skull were glued together. Finally, an agar-based mixture mimicking the gray matter was prepared with respect to the procedure described in Lacik et al. (2020), poured into the skull, and cooled to room temperature.

**Figure 4:**
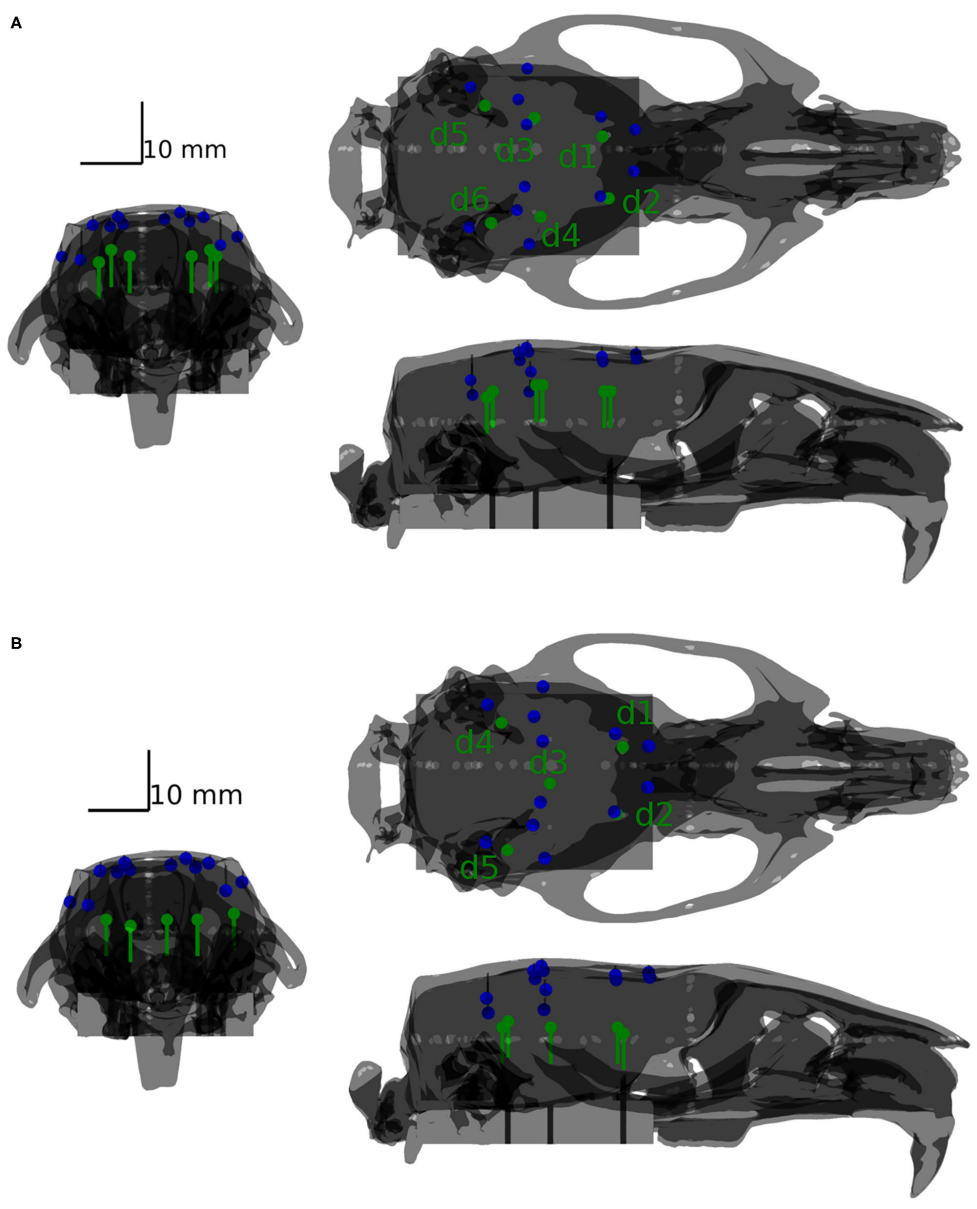
Positions of dipoles (green) with respect to the positions of the sensors (blue) depicted for the shallow **(A)** and deep **(B)** phantoms.

Two configurations of testing dipole positions were finally chosen for the validation purpose: a phantom containing six shallow dipoles and a phantom containing five deep testing dipoles. The two mentioned configurations are depicted in Figure 4. For more details of the positioning of the dipoles, please refer to the Supplementary Material. The shallow phantom simulates activation in the cortex, while the deep phantom is dedicated to testing how well deeper sources may be reconstructed.

A generator providing a harmonic signal with a frequency of 1 kHz was gradually connected to the excitation dipoles, and sensing electrodes were connected to the EEG amplifier. The following two test procedures were performed:

- The amplitude of the generator was set to 20 mV, and all of the dipoles were individually connected to the generator.
- The amplitude of the generator was varied in the range of 6–200 mV, and dipole d1 was excited.

After each test procedure, post-processing was applied to the electrode voltages obtained (Lacik et al., 2020). The first procedure was performed to test the localization algorithms and the second one to verify the operation of the phantom measurements in the linear regime at a test frequency of excitation of 1 kHz. Detailed information about the phantom fabrication and testing may be found in Lacik et al. (2020).

Once the experimental part was finished, the following analytical pipeline was applied to the phantom data. A CT scan of the fabricated phantom was acquired and spatially co-registered with the phantom geometry to precisely define the coordinates of the source dipoles. The head model and source model were prepared in the same way as described in section 3.1. Recorded time series of 1 min in length were divided into 1 s long segments. The two inverse solvers DICS and eLORETA described in section 3.2 were utilized to estimate dipole moments in the agar volume. Based on the CT image, the ED1 error was calculated for both inverse methods, see section 3.3. The distribution of the estimated dipole moments was printed over the CT image to ensure appropriate localization of the dipoles, see **Figures 7**, **8** in section 4.2.

There are several phantom model specific settings to be considered. Firstly, the source model was twice as sparse in physical units due to the up-scaling of the phantom. Nevertheless, the relative density of the sources was preserved across all of the evaluation methods. Secondly, the source model occupied the whole agar compartment. Therefore, areas were not distinguished between, and all of the positions within the brain compartment were considered as potential sources. The presence of dipoles, connecting coaxial cables, and a small amount of supporting glue were neglected in the forward model, see Figure 3.

### 3.5. ASSR Experiments

The Auditory Steady-State Response paradigm (ASSR) is a good tool for studying brain responses to periodically presented auditory stimuli of different frequencies. The ASSR constitutes a periodic phase-locked electroencephalographic response to periodic auditory stimulation, which allows inverse solvers to be evaluated *in vivo*, as the brain response pathway is anatomically well-defined. For the purpose of validating different solvers, we used data from our laboratory collected in preclinical research on ASSR paradigm in rats. In this experiment, we exclusively used baseline records from a total number of 71 rats.

Seven days before the EEG recording, Wistar male rats (280–300 g) were stereotactically implanted with 14 gold-plated electrodes (Mill-Max) under general isoflurane anesthesia (2.5% concentration). Electrodes were implanted onto the surface of the cortex in homologous frontal, parietal and temporal regions of the right and left hemispheres. Coordinates were taken from the Paxinos rat brain atlas (Watson and Paxinos, 2013). Their positions are listed in Figure 1A. The reference electrode was implanted above the olfactory bulb and the ground electrode subcutaneously in the occipital region. All of the electrodes were fixed to the skull with dental cement. Subsequently, the rats were housed individually in cages in order to prevent them from biting the implant, handled twice daily and touched on the implant in order to habituate them to the manipulations. The day before EEG recording, a 14-pin connector was plugged into the electrodes and fixed with dental cement under a short-term total isoflurane anesthesia (an already established procedure in our laboratory).

For 3 consecutive days prior to recording, the rats were habituated to the acquisition room and to the auditory sham stimulation (a short 10-min session with ASSR auditory paradigm without EEG recording). For the EEG recordings, the animals were connected to the EEG amplifier system in their home cages. A 15-min (5-min habituation, 10-min baseline) recording session was performed before the auditory experiment. After the 15-min baseline EEG recording, ASSR data acquisition was performed at 90 dB with single clicks and clicks at the frequencies of 10, 43, and 90 Hz and amplitude modulated (AM) tones at the frequency of 43 Hz via a stereo speaker sound system placed on both sides of the animal's cage. Each click/AM train lasted 1 s (except for the single clicks) with randomized inter-stimulus intervals set between 2 and 4 s. The protocol of our ASSR experiment was based primarily on the study of (Leishman et al., 2015). The frequencies and modulation types were presented in pseudo-randomized runs in two 20-min sessions separated with a 10-min pause without auditory stimulation. A total of 120 trains of each frequency and each modulation type were presented, which took 65 min in total. The rats were able to move freely in their cages during all of the EEG recordings, while being connected to a data acquisition system. Each rat was recorded only once. All handling of animals was approved by the ethical committee for work with laboratory animals at the 3rd Medical Faculty of Charles University and the National Institute of Mental Health and was performed in accordance with Directive 86/609/EU.

### 3.6. Evaluation of *in vivo* Experiments

As described in the previous section, *in vivo* evaluation of the ASSR experiments was conducted. An analytical pipeline for a real evoked data evaluation was prepared in order to:

- Be as close as possible to the classical approaches of evaluating steady-state response EEG data experiments
- Ensure all necessary preprocessing steps for removing artifacts
- Appropriately statistically evaluate the obtained results from inverse solutions
- Provide information about the size of datasets sufficient for performing source localization analysis

#### 3.6.1. Preprocessing

Datasets from 71 subjects were obtained. Based on time stamps, from each dataset, 120 segments consisting of 1-s stimuli intervals of 43 Hz clicking and a previous 1-s prestimulus interval as a baseline signal were extracted. Several preprocessing steps were performed in the order that they appear in the following text. These ensured minimization of the influence of artifacts on the obtained results. During the experiment, any outside interference, such as modification of the electrode system by experimenters, was manually marked as an artifact, and all stimuli intervals that fell within the artifact interval were excluded from further analysis. To detect broadband artifacts, the whole signal was bandpass filtered in a typical frequency range of between 110 and 130 Hz, where the EEG signal was assumed to have a significantly lower amplitude than the artifacts. Subsequently, a Hilbert transform followed by a z-transform was applied to the filtered signal. Firstly, the averaged envelope across all of the electrodes was calculated for each time sample. Secondly, the z-score value was calculated across all of the time samples of the averaged envelopes. Segments where the z-value exceeded the defined threshold were excluded from further analysis. The last artifact detection criterion was selection of the peak-to-peak amplitude limit of the signal, as a very high amplitude is considered to be an artifact and is often caused by the acquisition electronics. The last two mentioned preprocessing steps were taken from the FieldTrip recommendation of event-related potentials, (see the Fieldtrip tutorial). Finally, data were notch filtered at 50 Hz (DFT filter) and low-pass filtered with a cut-off frequency of 100 Hz (Butterworth, 6th order) and re-referenced to an average reference.

For the evaluation of *in vivo* experimental data, the three inverse solvers eLORETA, LCMV, and DICS were utilized. Sources in the late-latency response interval were estimated by time domain eLORETA and LCMV. Since the effect of the ASSR experiment in the entrainment segment is visible in a narrow frequency band around the frequency of the sound stimulation (**Figure 9B**), the frequency domain solvers eLORETA and DICS were chosen to estimate sources. Because of the poor results of sLORETA, this solver was excluded from the *in vivo* analysis. A cross-spectral density matrix for frequency domain eLORETA and DICS was calculated for the whole length of the entrainment part (**Figure 9A**) of the stimuli segment and the corresponding part of the prestimulus segment of the same length on the stimulus frequency, i.e., 43 Hz. Beside the evoked 43-Hz activity, which started to be prominent approximately 400 ms after the start of the stimulus, segments of early-latency (0–150 ms) and late-latency response (150–400 ms) also appeared in the first half of the stimulus interval. For validation purposes, two source reconstructions were calculated: late-latency response interval and ASSR entrainment interval at a frequency of 43 Hz, see **Figure 9A**. The corresponding length of the prestimulus signal was taken as a contrast condition. Filters for source localization were computed based on concatenated individual parts of the prestimulus and stimulus intervals, and these filters were used for source localization of the prestimulus and stimulus intervals separately. NAI was calculated for both solvers as a relative change of source activity, see Equation (7). The regularization parameter for the DICS solver was obtained using the same procedure as for LCMV, and eLORETA was regularized as in the surrogate evaluation, i.e., leave-one-out cross-validation. Our electrode system in the version presented in this study does not provide LFP measurements. Nevertheless, to be consistent with the previously mentioned two evaluation approaches, we also localized activity from the subcortical area of the brain. For that purpose, the early-latency response segment between 5 and 9 ms was chosen to localize a thalamic component that should be observable in data and falls within this time window, see **Figure 10A** for EEG traces and **Figure 10B** for a voltage map. An auditory evoked potential waveform attributed to be of thalamic generation is appeared on the cortical surface of the rat's brain with 7-8 ms latency from the stimulus onset, as was established by several other studies (Shaw, 1991, 1993; Barth et al., 1995). To localize the thalamic component, a time-domain eLORETA was utilized and the power of the estimated activity was directly subjected to the same statistical evaluation as the source localization of the late-latency response and ASSR entrainment segments. The data for the thalamic component source localization were preprocessed in the same manner except for the final filtering, where the data were bandpass filtered (Butterworth, 6th order) between 100 and 400 Hz, since we assumed that this thalamic component appears in high frequencies.

#### 3.6.2. Statistical Evaluation

For statistical evaluation, a cluster-based permutation statistical test (Maris and Oostenveld, 2007) was performed to check the so-called family-wise error rate. For each subject, NAIs in each source position were averaged over the trials. During the stimulation, the signal on each electrode showed an increase in power in the examined frequency band; hence, the whole brain showed an activity increase. For this reason, statistically testing NAIs against zero would not be specific enough. Therefore, the average NAI over the whole brain for each subject was taken as a contrast condition. Subsequently, these two conditions were randomly divided into two different subsets, where each subset comprised a random part of both of the experimental conditions. A paired sample one-sided t-test was calculated in each source position, and clusters were constructed. A cluster was defined as a volume of adjacent source positions where the t-values exceeded a predefined threshold (corresponding to *p* < 10^−3^ for late-latency and ASSR entrainment and *p* < 10^−9^ for the thalamic component). This process was repeated 1,000 times, and in each iteration cluster, the highest sum of t-values was utilized for constructing cluster-level statistics. Finally, clusters found in non-permuted data were compared with cluster-level statistics. Clusters with a Monte Carlo *p*-value lower than the 0.05 significance level of the cluster statistics were marked as significant, see Maris and Oostenveld (2007).

## 4. Results

This section summarizes the results obtained by the methods described above.

### 4.1. Evaluation of Surrogate Data

Based on the methodology (see Figure 2), surrogate data on the electrodes were created by simulating a 100-sample-long Gaussian noise signal on a source level and propagating to the sensor level via the lead field matrix. A simple model of additive noise to sensor level data provided the desired levels of SNRs for testing the inverse solvers under different data quality conditions. Two types of error distance measurements, ED1 and ED2, were performed. Table 2 summarizes the average ED1 and ED2 values for each inverse solver and for each of the four levels of SNR. The results in this table are averaged over the simulated dipole moment direction, and ED1 is expressed as a Euclidean distance. See the Supplementary Material for a detailed version of this table with separate ED1 in the *x*, *y*, and *z* directions. These results indicate that eLORETA performs best with the lowest ED1 under all of the data quality conditions. The LCMV solver performed worse in terms of the ED1 criterion, but the ED2 criterion in the LCMV case decreased rapidly with increasing SNR. Therefore, it is obvious that source localization by LCMV is more focused than that of eLORETA in the single dipole simulation scenario. Surprisingly, the sLORETA inverse solution was not sensitive to data quality. It is difficult to determine what part of the model causes poor localization of sLORETA, but a mean error distance of almost 4 mm over all of the SNRs makes the sLORETA solver unsuitable for the required purposes.

**Table 2:** Mean and standard deviation of the averaged error distance measurements ED1 and ED2 over all of the positions of the source model and dipole moment directions for LCMV, sLORETA, and eLORETA inverse solvers and SNR levels 5, 10, 15, and 25 dB.

**Inverse** **solvers**	**Error** **measurement**	**SNR (dB)**			
		**5**	**10**	**15**	**25**
sLORETA	ED1 (mm)	3.9 ± 2.5	3.9 ± 2.5	3.9 ± 2.5	3.9 ± 2.5
	ED2 (-)	94.9 ± 37.6	89.3 ± 38.2	87.4 ± 38.6	86.7 ± 39.0
eLORETA	ED1 (mm)	1.3 ± 1.6	0.8 ± 1.3	0.4 ± 1.0	**0.2** **±** **0.7**
	ED2 (-)	109.8 ± 42.7	106.0 ± 43.1	104.7 ± 43.2	103.8 ± 43.0
LCMV	ED1 (mm)	3.0 ± 2.2	2.1 ± 2.1	1.5 ± 1.9	1.1 ± 1.6
	ED2 (-)	105.1 ± 43.6	71.8 ± 37.4	40.9 ± 28.6	**9.5** **±** **9.9**

*Bold values depict the minima of ED1 and ED2 error measurements*.

**Figure 5:**
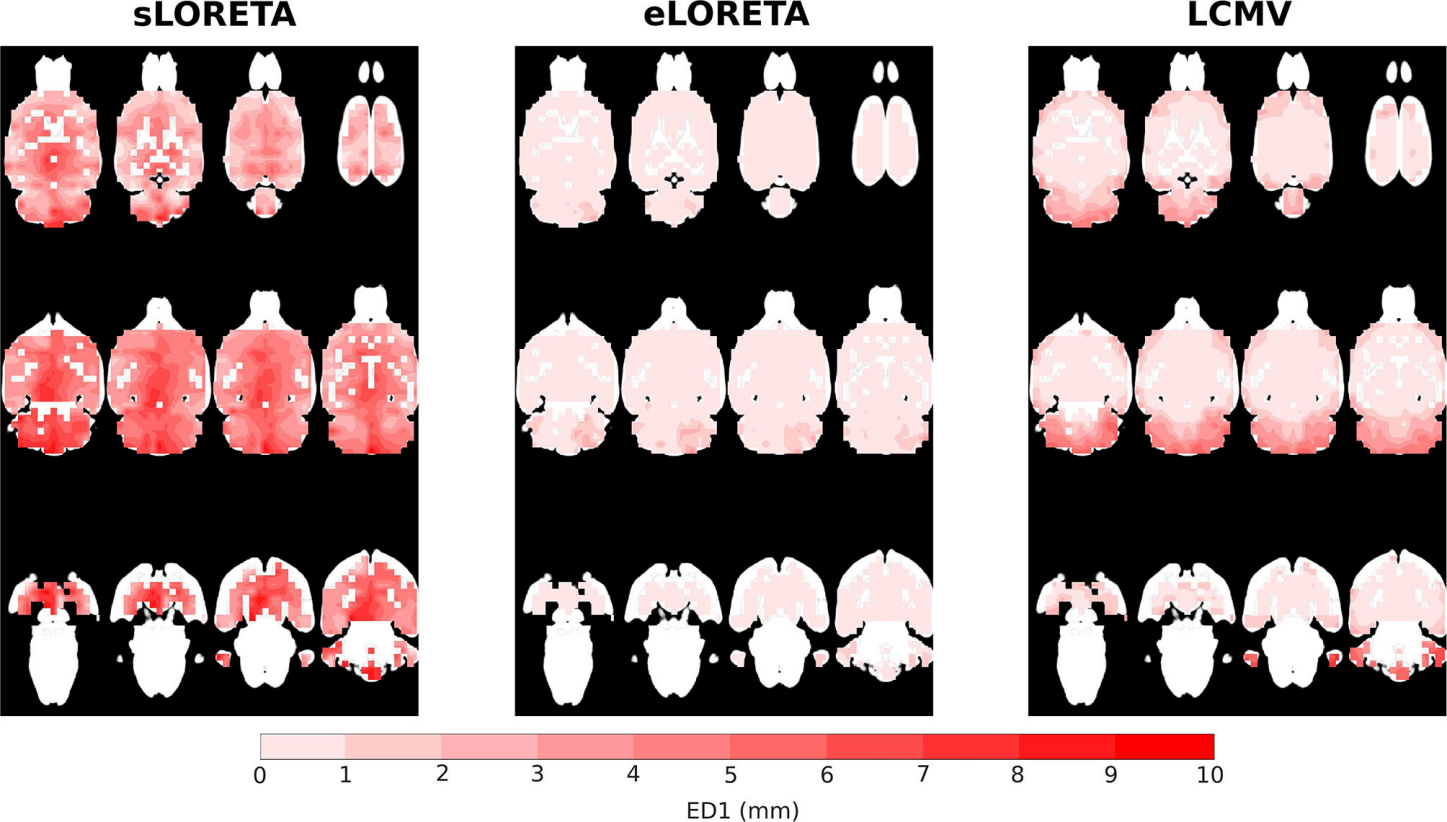
ED1 values interpolated on MRI scan axial slices for sLORETA, eLORETA, and LCMV inverse solvers with SNR 25 dB.

The distribution of localization error ED1 shows the limits of this source localization approach and the maps provide an indication of how trustworthy the localized sources are, if they are expected to be active in a specific location, see Figure 5. Based on the obtained maps, the highest ED1 values are concentrated in parts of the brain that are less visible for the electrodes, i.e., electrode coverage above these brain areas is poor. Specifically, the cerebellum, including 80% of the total number of neurons seems to be the most sensitive to the ED1 type of error. The peripheral frontal part of the brain exhibits higher values of ED1. Comparing solvers across the same SNR of 25 dB, eLORETA shows ED1 values higher than 1 mm only in the cerebellum, whereas LCMV also shows them in the mentioned frontal part of the brain. The sLORETA ED1 map shows a tendency for high ED1 values in the central part of the brain and bias in the *z*-axis. Surprisingly, neither eLORETA nor LCMV show ED1 bias in the *z*-axis.

**Figure 6:**
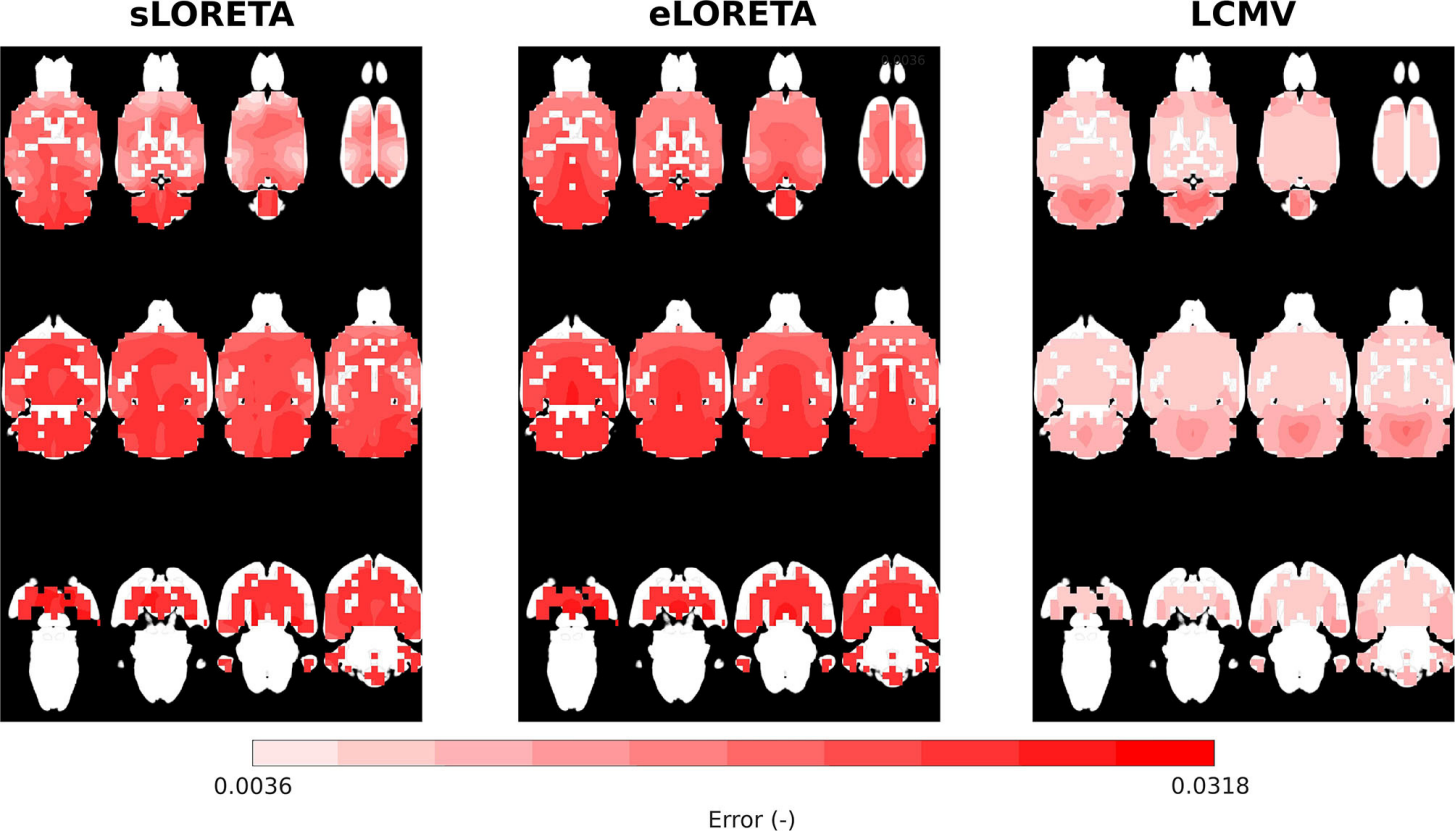
Reliability maps interpolated on MRI scan axial slices for eLORETA, LCMV, and sLORETA inverse solvers with SNR = 25 dB.

The reliability maps expressed the trustworthiness of the estimated solution in a particular location. It may be seen that ED1 and the reliability maps partially correspond to each other, especially in the LCMV case, where the estimated solution is focused, see Figure 6. The distribution of reliability map error values for eLORETA corresponds to a blurry estimated solution, which appears across all SNRs. The obtained reliability maps show a minor error bias in the *z*-axis.

Overall, this methodology gives a solid idea of the behavior of the whole model and allows an indication of its weaknesses and advantages in applications with real data. Based on these findings, it will be possible to continually test and improve new versions of the electrode system and numerical model. The obtained numerical results correspond well to those measured on the fabricated phantom.

### 4.2. Evaluation of Phantom Measurements

In the previous subsection, numerical simulations evaluated the spatial distribution of localization errors in the case of the same generative and inverse models. In other words, the testing surrogate data were generated by the same head model, which was consequently used for source localization. Here, the fabricated phantom tested the inverse techniques in cases where the generative (real measurement) and inverse (numerical simulation) models were different. Small differences between the brain and a forward numerical model are always present in real experiments. The main contribution of measurements on the phantom was to simulate these circumstances. This subsection summarizes the most relevant results obtained in the phantom experiment.

A simple, realistic phantom based on agar, electrodes, and coaxial current dipoles was fabricated. The coaxial dipoles were found to sufficiently approximate the numerical assumptions, and the whole domain could be consequently numerically modeled with sufficiently high precision for solving ESI. Since the deviations of the numerical model from the fabricated phantom were not crucial, the phantom approach may be used for the validation of electrode implants.

**Figure 7:**
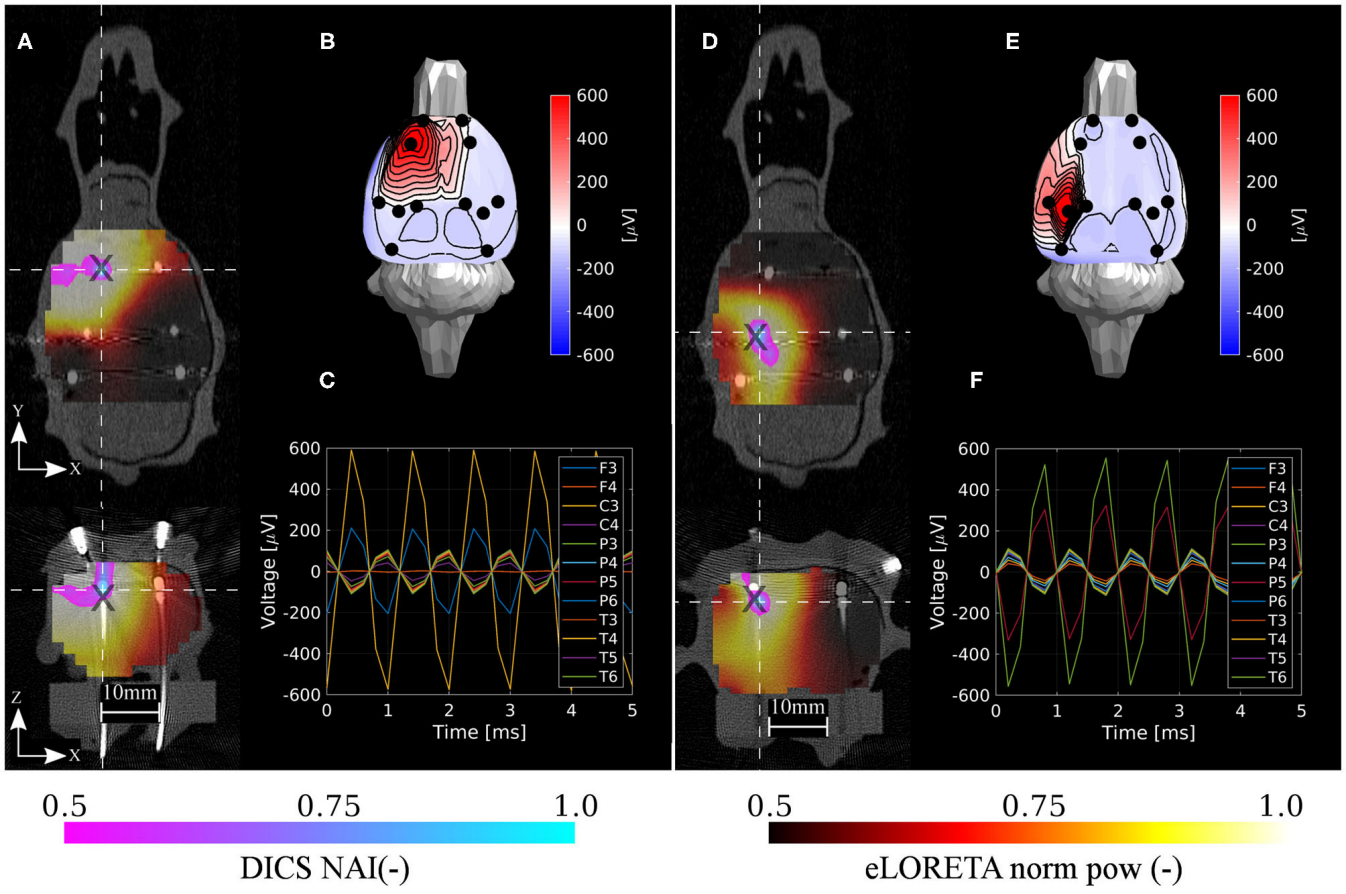
Localization results for the shallow phantom. Estimated neural activity indices by the DICS and eLORETA algorithms **(A,D)**, dipole excitation measured on sensors **(B,E)**, and time courses on sensors **(C,F)** for the two selected reference source dipoles d1 and d3, respectively. Here, d1 and d3 were associated with the best and the worst localization accuracy, respectively. The source estimations are interpolated onto the CT volume image and depicted in axial and coronal planes **(A,D)**. The intersection of both planes is located at the maximum of the estimated NAI. Coordinates of reference dipoles are marked with black crosses. Coordinates of DICS NAI maxima are marked with white dashed crosses.

**Figure 8:**
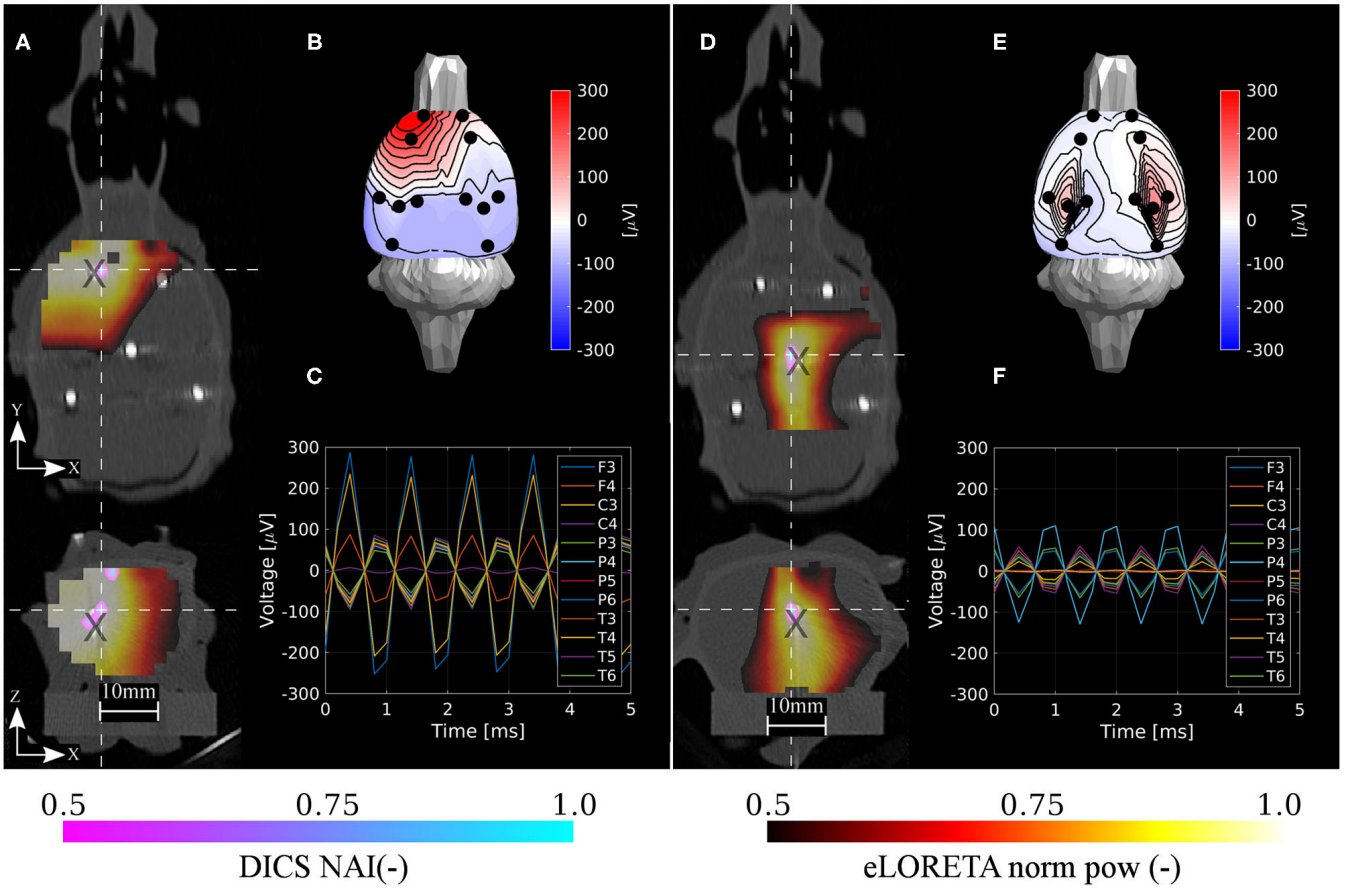
Localization results for the deep phantom. Estimated neural activity indices by the DICS and eLORETA algorithms **(A,D)**, dipole excitation measured on sensors **(B,E)**, and time courses on sensors **(C,F)** for the two selected reference source dipoles d1 and d3, respectively. Here, d1 dipole was a deeper version of d1 dipole in the shallow phantom and d3 dipole was placed approximately to the location of the found thalamic activity in the ASSR experiment. The source estimations are interpolated onto the CT volume image and depicted in axial and coronal planes **(A,D)**. The intersection of both planes is located at the maximum of the estimated NAI. Coordinates of reference dipoles are marked with black crosses. Coordinates of DICS NAI maxima are marked with white dashed crosses.

The eLORETA and DICS inverse algorithms both performed well in localizing the shallow and deep excited dipoles once appropriate regularization was applied (Jonmohamadi et al., 2014). Surprisingly, localization error ED1 was not primarily found in the *z*-axis in the case of the shallow phantom. Hence, only a slight depth bias was observed in localizing the testing dipoles. The DICS precision was found to be much better than that of the eLORETA. The DICS algorithm resulted qualitatively in very high precision (spatially specific maps) since it also utilizes the sensor cross-spectral matrix, and enough data were inputted for its estimation, see Figures 7, 8. It should be noted that similar maps to DICS may be achieved with eLORETA by setting a higher threshold for the colormaps. Here, the upper half of the range of both methods was chosen.

In order to assess the system in the sense of depth bias, the averaged errors in separate directions were computed. In the case of the shallow testing dipoles, the averaged errors across dipoles and methods in X, Y, and Z directions were 0.17, −0.17, 0.33 mm, respectively. In the case of the deep testing dipoles, the averaged errors across dipoles and methods in X, Y, and Z directions were 0.0, 0.4, 0.8 mm, respectively. Hence, in both cases the averaged errors in the Z direction were positive (toward the electrodes) and approximately two times higher than the other directions. The maximum positive errors in the shallow and deep dipoles in the Z direction were 2 and 3 mm, respectively.

**Figure 9:**
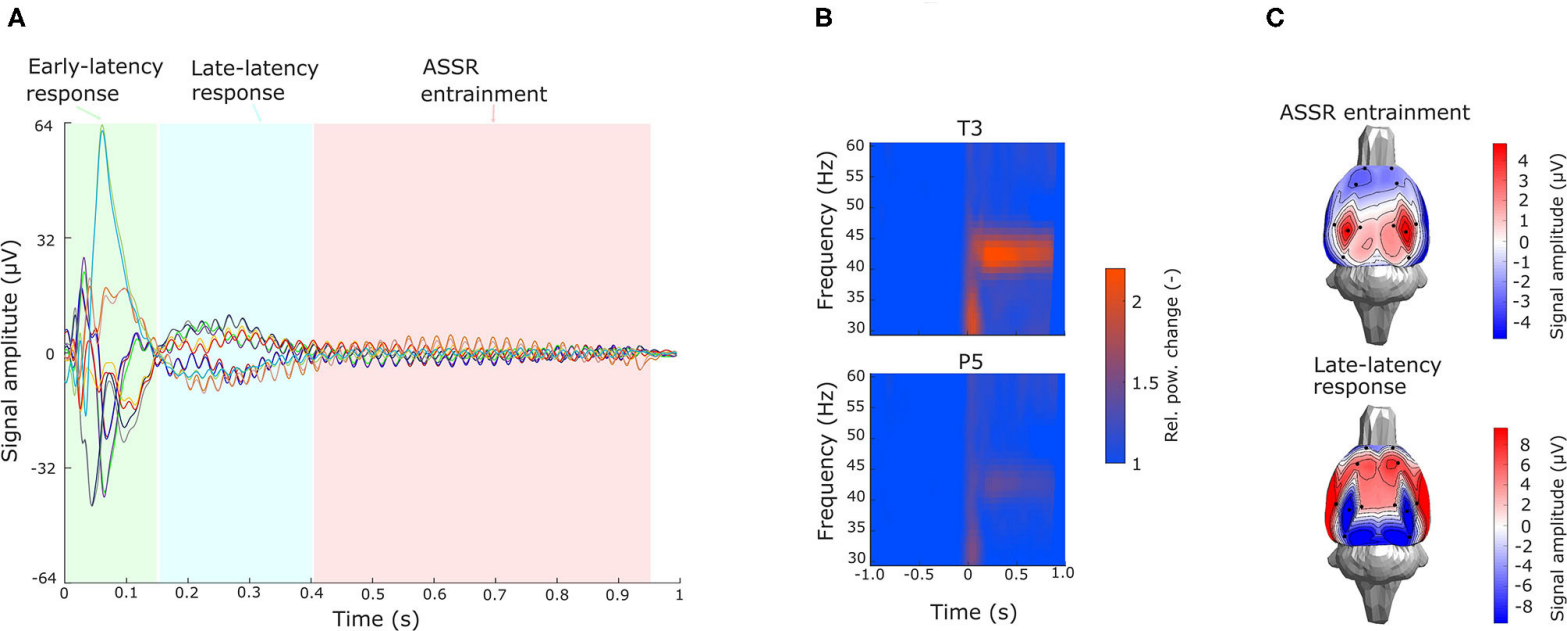
Preprocessed ASSR data. **(A)** Butterfly plot of a grand averaged evoked potential with stimuli onset at 0 s across all electrodes. Stimulus interval is divided into early-latency response (0–150 ms), late-latency response (150–400 ms), and 43-Hz ASSR entrainment (400–950 ms) segments. **(B)** Grand averaged time–frequency analysis for frequency bands ranging from 30 to 60 Hz with 1-Hz resolution on electrodes T3 and P5. Values are normalized by the mean value of the prestimulus segment in each frequency band. **(C)** Grand averaged signal amplitude topography maps for ASSR entrainment and late-latency segments.

The results are in excellent correspondence with the results of the numerical evaluation. The localization accuracy is summarized in Table 3. In shallow sources, both inverse algorithms resulted in the same accuracy of 1.6 mm on average, which is clearly related to the ED1 maps in Figure 9. In terms of accuracy, close ED1 values were obtained for both inverse algorithms and two regularization approaches. Therefore, the source distributions provided by both methods overlaid well. In the case of the deep testing dipoles, both algorithms again showed a similar accuracy of 2.6 mm on average. These results indicate that the 12-electrode system facilitates ESI and it is a solid base for the development of future ESI dedicated implants.

**Table 3:** Error distances (ED1) for all six shallow and five deep dipoles for both inverse algorithms eLORETA and DICS.

	**Shallow dipoles**						**Deep dipoles**				
	**d1**	**d2**	**d3**	**d4**	**d5**	**d6**	**d1**	**d2**	**d3**	**d4**	**d5**
	elor	elor	elor	elor	elor	elor	elor	elor	elor	elor	elor
	dics	dics	dics	dics	dics	dics	dics	dics	dics	dics	dics
ED1	1.0	1.4	3.7	1.0	1.4	1.0	1.7	2.8	2.4	3.7	2.2
(mm)	1.0	1.4	1.4	1.0	1.4	1.0	3.3	2.8	2.4	3.2	1.0
*X*	0.0	0.0	1.0	1.0	−1.0	0.0	-1.0	2.0	-1.0	-3.0	2.0
(mm)	0.0	0.0	1.0	1.0	−1.0	0.0	1.0	-2.0	-1.0	3.0	0.0
*Y*	0.0	−1.0	-3.0	0.0	1.0	0.0	1.0	2.0	1.0	-1.0	0.0
(mm)	0.0	−1.0	1.0	0.0	1.0	0.0	1.0	0.0	1.0	-1.0	0.0
*Z*	1.0	1.0	2.0	0.0	0.0	−1.0	1.0	0.0	2.0	-2.0	1.0
(mm)	1.0	1.0	0.0	0.0	0.0	−1.0	3.0	2.0	2.0	0.0	-1.0

### 4.3. Evaluation of *in vivo* Experiments

To test the viability of the setup for ESI on real data, a large dataset of cortical recordings from the ASSR experiment was used. The artifact detection approach described in the section 3 was applied to identify and reject segments contaminated with biological and technical artifacts, whereby obtaining the complex evoked potentials shown in Figure 9A (grand average on electrodes T3 and P5). Each 1-s fragment was divided into three segments. The first segment (early evoked potentials), lasting from 0 to 150 ms, exhibits fast oscillations with a complex behavior over all of the electrodes. The late-latency response, lasting from 150 to 400 ms, shows a slow wave with a peak around 350 ms, and a 43-Hz oscillation coming up over all of the electrodes. The third segment contains ASSR entrainment lasting from 400 ms until the end of the signal fragment and exhibits mostly 43-Hz oscillations, see Figure 9A. These oscillations are considered to originate in the auditory cortex. This is supported by the time–frequency analysis, where electrodes located near the auditory cortex (T3–T6) contain a significant frequency component in the band corresponding to the stimulus, while on the other electrodes, this component is not so prominent, see Figure 9B. For source analysis, late-latency response, ASSR entrainment and thalamic component segments were considered. Late-latency response and thalamic component segments were analyzed in the time domain and in a broad frequency band, whereas ASSR entrainment was analyzed in the frequency domain, since the signal contains one strong frequency component. Based on the averaged topographical maps calculated over the defined segments (Figure 9C), in the case of ASSR entrainment, source localization with two dipoles may be assumed, whereas in the case of late-latency such an assumption could not be made. Source localization of the thalamic component should result in a single activated area located in the thalamus.

**Figure 10:**
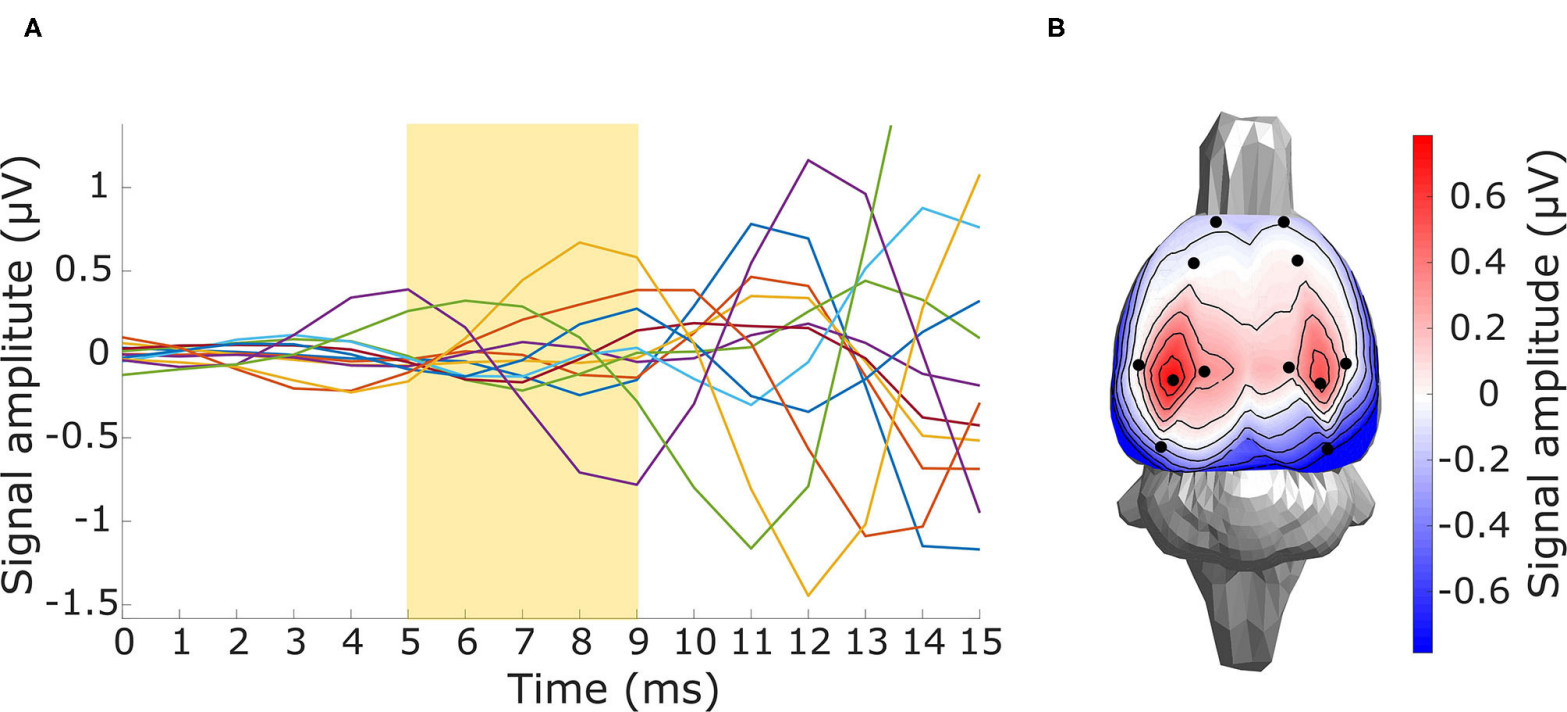
Preprocessed thalamic component data. **(A)** Butterfly plot of a grand averaged evoked potential for the first 15 ms after the stimuli onset with the yellow highlighted segment (5–9 ms) of the assumed thalamic component. **(B)** Signal topography of the yellow highlighted thalamic component segment.

**Figure 11:**
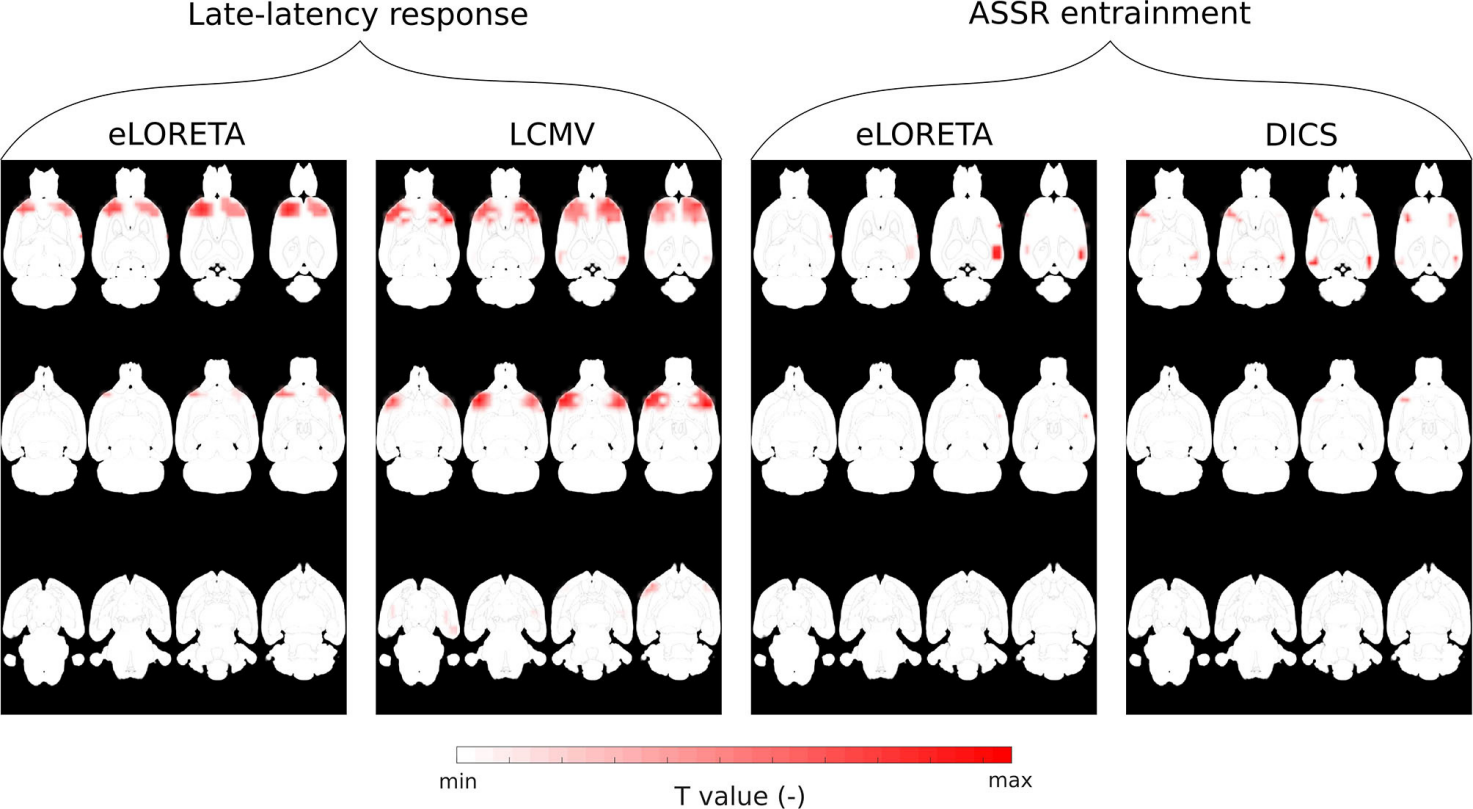
Significant clusters obtained by non-parametric cluster-based statistical testing expressed in normalized T values and interpolated on MRI scan axial slices. Statistical maps for late-latency segments were obtained by eLORETA and LCMV in the time domain, whereas maps for the ASSR entrainment segment were obtained by eLORETA and DICS in the frequency domain (43 Hz).

**Figure 12:**
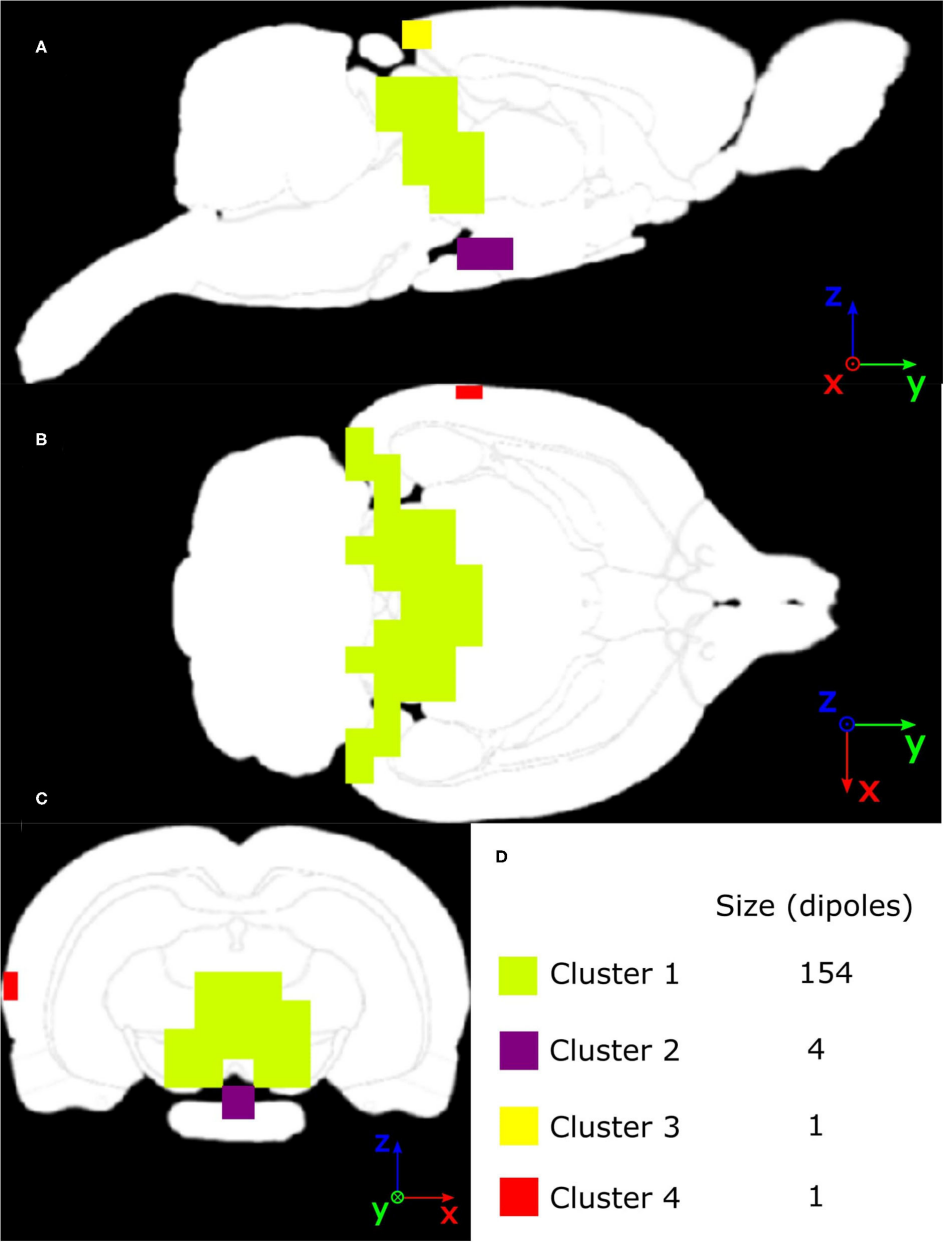
Masks of four significant clusters of thalamic component source localization obtained by non-parametric cluster-based statistical testing interpolated on MRI scan of **(A)** Sagittal slice, **(B)** Axial slice, **(C)** Coronal slice, with an intersection in the global maximum of T values located in cluster 1.

Non-parametric cluster-based statistical evaluation of the obtained results of source localization on the large dataset revealed the parts of the brain that were activated over a specific period in the response. Figure 11 shows that in the late-latency response segment, the main activated areas were in the frontal part of the cortex, largely overlapping the pre-motor cortex, for the eLORETA and the LCMV inverse solvers. In the frequency domain, source localization analysis revealed activity in the auditory cortex (see Figure 11), as expected. Furthermore, the DICS method also indicated small activations in the frontal part of the cortex, which were not indicated by eLORETA. In the case of eLORETA, significant sources were located almost unilaterally in the auditory part of the cortex with minor activation present also in the left auditory part of the cortex. Source localization of the thalamic component, defined in the time window 5 - 9 ms after the stimuli onset (see Figure 10A for EEG traces and Figure 10B for signal topography), by time-domain eLORETA resulted in four significant clusters, see Figure 12. The largest cluster consists of 154 dipoles and extends widely around the posterior area of the thalamus and inferior colliculus. The second largest cluster consisting of four dipoles forms a small defined area at the central base of the brain. Clusters three and four are each formed by a single dipole.

Overall, source localization of the ASSR entrained part of the evoked potential strongly indicates that the 12-electrode cortical EEG system allows shallow sources of the brain to be reliably localized when enough data are provided. The original finding of activity in the frontal part of the cortex also points to the possible benefit of this method for further exploring brain activity from EEG without a common pickup phenomenon typical when data are processed, and results interpreted at the electrode level. The significant clusters found in the source localization of the thalamic component suggest that the electrical activity not only from the shallow sources but also from deeper areas of the brain may be obtained, which is also in correspondence with the results obtained from the surrogate and phantom data measurements.

Since ASSR activities were evaluated using a large amount of data, the question arises of how much data is needed to obtain a similar quality of source localization of evoked potentials using the present methodology. To answer this, the spatial correlations of the grand averaged source estimate when using data from fewer subjects and the grand average source estimate of the entire dataset were computed. These are shown in the figure included in the Supplementary Material as a function of the number of subjects used. It may be seen that correlations in the order of 0.9 may be obtained for data from 71 subjects; data from approximately 30–40 subjects are needed for both inverse methods. While this is heuristic, it gives an indication of the amount of data needed to apply the approach successfully.

## 5. Discussion

This paper presents a 12-electrode rat cortical EEG system and studies its ability to localize EEG sources in cortical and subcortical regions. Due to the complexity of the source localization problem, the system was validated using data from a computational model and from a physical phantom of the rat's brain, as well as real cortical EEG recordings from an ASSR experiment. It has been shown that the proposed 12-electrode cortical EEG system allows rat brain activity to be reliably reconstructed from the potential recordings obtained using the electrode system. The evaluation strategy proposed here may also be used in the evaluation of future electrode systems. The viability of this system for source localization has two important implications: the currently used EEG system is a solid base for optimization toward an ESI dedicated system, and the existing database of animal EEG data may be analyzed in terms of the ESI method with the performance and limitations found in this study.

Simulated mean error distance ED1 over the whole brain was lowest in the eLORETA solution through all of the SNRs. The lowest and highest localization errors across DICS and eLORETA methods including the cerebellum area were 0.2 and 3.0 mm for 25 and 5 dB SNR, respectively. The ED1 was inhomogeneously distributed in the rat brain volume and the highest ED1 values were found in the cerebellum area, which was not covered by the sensing electrodes. In human ESI research, a recent paper (Song et al., 2015) reported a mean simulated localization error of 3.65 and 1.9 mm for 20dB SNR for 16- and 10-20-electrodes systems, respectively. Here, considering an approximate ratio of human and rat brain of 10:1, the simulated mean errors in rats corresponds to the accuracy obtained by a comparable number of electrodes in humans. In the fabricated upscaled phantom, mean error distances of 1.6 mm and 2.6 mm were obtained across the testing dipoles for shallow and deep dipoles, respectively. The maximal error in the phantom measurements was 3.7 mm, which corresponds to an error of 1.9 mm on the scale of a rat's brain. These results ensure that the resolution of the current method was sufficient to distinguish between functional areas of the rat's brain. The results obtained in the ASSR experiment are in a correspondence with the available literature and are discussed in detail below.

The evaluation of the cortical EEG system presented here has certain limitations. It is difficult to interpret the ED1 error reported in Table 2 in terms of mean and standard deviation across the tested dipoles since this error is not homogeneously distributed in the brain volume. Therefore, the ED1 maps depicted in Figure 5 also need to be considered when addressing the ED1 error. The ED1 error does not consider spurious activation of the dipoles, so the reliability maps shown in Figure 6 should be further consulted for a better understanding of the error properties. In this study, the information from the ED1 table, ED1 maps, and reliability maps are consistent, which is one of the key conclusions. The main phantom limitation is relatively fast expiration of the agar gelatin simulating the brain tissue. Furthermore, the iterations of the phantom development were slowed down by the need for a CT scan, which could not have been performed in-house. The scan was necessary for precise localization of the testing dipoles. The agar-based phantom had to be measured at a frequency of 1 kHz to prevent non-linear phenomena on the electrode–agar interface at lower frequencies. In the case of the ASSR experiment, the sources found should be further validated by deep electrodes simultaneously recorded with the cortical ones. An implant capable of concurrent local field potential and cortical EEG recording for validation purposes is currently under development. Future electrode locations will be optimized using a method based on singular value optimization of lead field matrices.

Upon evaluation of the methodology of the phantom measurements, a slight depth bias was observed in localizing the testing dipoles. Both shallow and deep versions of the testing dipoles showed an approximately two times higher error in the Z direction compared to the other directions on average. However, a maximum positive error in the Z direction was 3 mm in the case of the deep dipoles. Because the phantom underwent an approximately twofold enlargement, in the rat head coordinate system the maximum observed error in the Z direction would be approximately 1.5 mm, which is still enough to reliably localize and register deep sources. It may be speculated that the improvement in the depth bias with respect to a previous study (Yang and Jiang, 2013) is due to a small but non-zero variance of coordinates in the *Z* direction across the electrodes. The previous study (Yang and Jiang, 2013) assumed a brick-shaped phantom with a flat distribution of the electrodes. Experimental justification of this phenomenon is out of the scope of this paper. However, it will be studied in the future, along with further optimization of the electrode positions.

Empirical EEG data during ASSRs in the rat trials were used to compare the distributed nature of the source localization. Identifying the sources underlying key events of information processing such as auditory evoked potential (AEP) peaks or oscillatory brain activity such as ASSR-entrained spontaneous gamma oscillations has been the objective of many research studies. To evaluate the source localization method, the EEG dataset was separated into three distinct epochs: early-latency response, late-latency response, and ASSR entrainment. The latter two were localized as a whole, from the early-latency epoch we only selected a time window of 5–9 ms. DICS and eLORETA were used to compute the sources underlying the electrophysiological activity. A late-latency peak of around 300 ms as well as its topology distributed broadly over the midline scalp were also observed in other rat AEP studies (Hurlbut et al., 1987; Ehlers et al., 1994). A late-latency component in rats occurred in the latency range of the human auditory P300 and shared with them several other characteristics (Shuhei et al., 1993). However, despite apparent similarities, the human P300 latency component is elicited in the process of decision making and is thought to reflect processes involved in stimulus evaluation or categorization (Polich, 2007). In terms of source localization, the P300 peak in humans has been repeatedly attributed to activation of the prefrontal cortex, parietal cortex, temporal lobe, and anterior cingulum (Tarkka and Stokic, 1998; Sabeti et al., 2016). The present study found major and spatially distinct activation of the primary motor cortex in a rat using the eLORETA and DICS approaches. We hypothesize this activation to be a consequence of the rat's behavioral reaction, as it convulsively freezes during the presentation of stimulus. Temporal processing of auditory stimuli may be investigated by analyzing ASSRs in the EEG. ASSR is observed when periodically presented auditory stimuli produce electroencephalographic entrainment—afferent neurons in the central auditory system synchronize their firing patterns to a particular phase of these stimuli and approach the same frequency (Picton et al., 2003). eLORETA and DICS were both able to pinpoint the auditory cortex, which is in strong accordance with many human studies (Herdman et al., 2002; Reyes et al., 2005; Halder et al., 2019). Therefore, activation of the primary sensory regions involved in ASSR auditory processing appears to be consistent across species. Localization of subcortical areas in a precisely defined time window of 5–9 ms by time domain eLORETA resulted in capturing the activity in the area of the thalamus, inferior colliculus and also in the lower part of the brain anatomically corresponding to the superior olivary complex. The superior olivary complex, inferior colliculus and medial geniculate nucleus, which is part of the auditory thalamus located in its posterior part, are areas present at the neuronal relay from the cochlea to auditory cortex (Mamach et al., 2018) and their activity is expected in the chosen time window (Shaw, 1993).

The main limitations of the studied EEG system are due to the need for invasive surgery. For example, more temporally placed electrodes cannot be implanted using the current technology. Therefore, covering the brain with electrodes in the *z*-axis is limited, even for future implants. The currently tested system was not originally developed for ESI and did not cover certain areas like the olfactory bulbs and cerebellum. Therefore, the number of electrodes and their positions may be further optimized to obtain more reliable localization.

The main advantage of the current system over the methods previously described in the literature is that it may be applied to freely moving animals. This facilitates more ecologically valid experiments with conscious and moving animals. Secondly, the cortical electrodes minimize the influence of low conductive and anisotropic skull to source localization. Hence, the solved inverse problem is better determined compared to previous methods, and more reliable solutions may be achieved. In this paper, a comprehensive evaluation procedure involving simulated data, a physical phantom, and an *in vivo* experiment was established and performed. This approach was chosen to inform experimental scientists about a particular system's properties from different perspectives to support the proper use of the system.

Overall, this paper establishes a strong basis for a reliable ESI methodology for preclinical rat model studies, which may serve as a powerful tool for the *in vivo* study of brain activity.

## Data Availability Statement

The raw data supporting the conclusions of this article will be made available by the authors, without undue reservation.

## Ethics Statement

The animal study was reviewed and approved by ethical committee for work with laboratory animals at the 3rd Medical Faculty of Charles University and the National Institute of Mental Health.

## Author Contributions

SJ and VK proposed the overall structure of the paper and performed evaluation steps. JL fabricated a rat head phantom and provided phantom measurement. CV and TP performed surgery, EEG recordings of *in vivo* experiment, and also provided expertise in EEG data evaluation. DK contributed to rat head forward modeling methodology. DW provided methodological supervision in inverse modeling and evaluation approaches. ZR provided forward modeling and phantom fabrication supervision. JH provided his expertise in the evaluation of inverse solvers as well as a paper structure and grammar check. All authors contributed to the article and approved the submitted version.

## Conflict of Interest

The authors declare that the research was conducted in the absence of any commercial or financial relationships that could be construed as a potential conflict of interest.
