# Supplementary material for: Electrical Source Imaging in Freely Moving Rats: Evaluation of a 12-Electrode Cortical Electroencephalography System
Source: Front Neuroinform. 2021 Jan 25;14:589228. doi: 10.3389/fninf.2020.589228 (PMC7868391; doi:10.3389/fninf.2020.589228)
Supplement: Supplementary file 1 [file Data_Sheet_1.ZIP › ESI_Rats_NUDZ_suppmat.pdf]

Supplementary Material

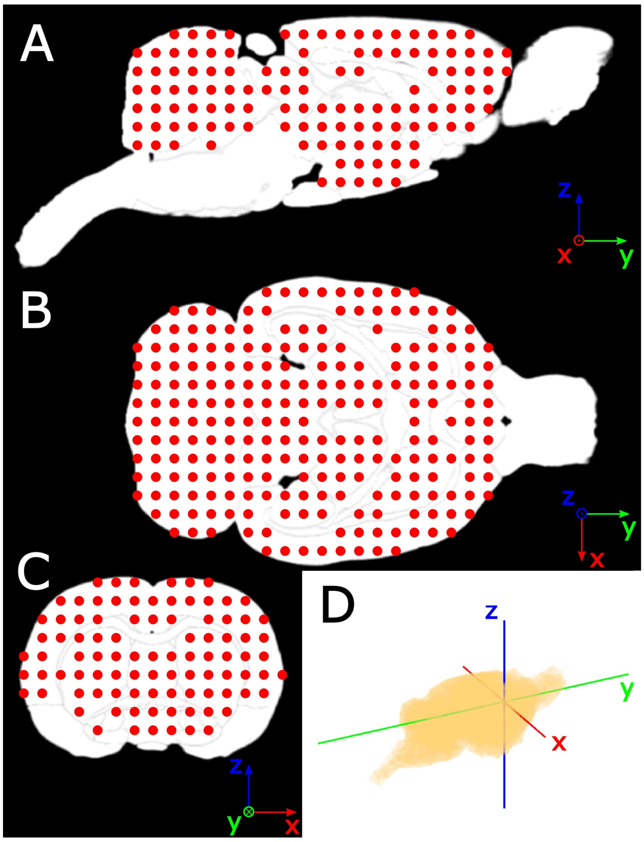

Figure S1. Visualized source model positions on MRI scan denoted as red circles. **(A)** Saggital slice. **(B)** Axial slice. **(C)** Coronal slice. **(D)** 3 orthogonal axes forming visualized slices with intersection in x = 0, y = 0, z = 2 mm in Waxholm space coordinate system.

Table S1. Mean and standard deviation of the error distance measurement ED1 in x,y, and z directions, and ED2, averaged over all positions of the source model, and dipole moment directions for LCMV, sLORETA, and eLORETA inverse solvers for SNR levels 5 dB, 10 dB, 15 dB and 25 dB.

Inverse solvers	Error measurement	SNR (dB)			
		5	10	15	25
sLORETA	ED1 x-axis (mm)	2.0±2.2	2.0±2.2	2.0±2.2	2.0±2.2
	ED1 y-axis (mm)	2.0±1.7	2.0±1.7	2.0±1.7	2.0±1.7
	ED1 z-axis (mm)	1.8±1.6	1.8±1.6	1.8±1.6	1.8±1.6
	ED1 euclid. (mm)	3.9±2.5	3.9±2.5	3.9±2.5	3.9±2.5
	ED2 (-)	94.9±37.6	89.3±38.2	87.4±38.6	86.7±39.0
eLORETA	ED1 x-axis (mm)	0.6±1.1	0.4±0.9	0.2±0.7	0.1±0.5
	ED1 y-axis (mm)	0.6±1.0	0.4±0.8	0.2±0.6	0.1±0.3
	ED1 z-axis (mm)	0.6±1.0	0.4±0.7	0.2±0.6	0.1±0.3
	ED1 euclid (mm)	1.3±1.6	0.8±1.3	0.4±1.0	0.2±0.7
	ED2 (-)	109.8±42.7	106.0±43.1	104.7±43.2	103.8±43.0
LCMV	ED1 x-axis (mm)	1.5±1.7	1.0±1.4	0.8±1.2	0.6±1.0
	ED1 y-axis (mm)	1.3±1.5	0.9±1.2	0.7±1.1	0.5±1.0
	ED1 z-axis (mm)	1.7±1.6	1.1±1.4	0.8±1.2	0.6±1.0
	ED1 euclid. (mm)	3.0±2.2	2.1±2.1	1.5±1.9	1.1±1.6
	ED2 (-)	105.1±43.6	71.8±37.4	40.9±28.6	9.5±9.9

Table S2. Distances of all testing dipoles in rat head phantom from all electrodes in millimeters.

	shallow dipoles						deep dipoles				
	d1	d2	d3	d4	d5	d6	d1	d2	d3	d4	d5
F3	7.5	13.0	16.9	21.2	25.4	28.2	10.2	14.8	19.2	25.9	29.8
F4	9.7	8.5	18.9	17.5	27.2	25.3	12.7	11.2	18.5	27.8	26.5
C3	5.8	14.1	11.4	19.3	19.7	25.2	9.2	15.3	15.6	20.4	27.0
C4	11.2	5.8	17.2	11.2	24.6	19.0	14.4	8.6	14.5	25.2	20.2
P3	13.9	18.9	5.6	16.1	10.5	18.1	16.9	19.5	11.7	12.0	20.6
P4	16.5	15.4	12.7	8.2	16.7	10.7	19.7	16.2	10.6	17.7	13.2
P5	15.7	22.2	5.6	19.7	8.1	21.1	17.9	22.5	13.9	9.7	23.5
P6	19.3	16.2	16.1	6.6	19.1	7.9	22.2	16.8	11.9	20.0	10.2
T3	16.4	24.8	8.1	24.1	9.1	25.6	17.0	24.4	16.3	9.5	27.7
T4	21.2	15.1	20.5	5.2	23.8	7.7	23.5	15.2	13.8	24.3	8.15
T5	22.7	28.6	11.5	23.9	3.8	22.2	23.6	28.0	16.7	4.4	24.2
T6	26.1	23.11	20.6	11.7	20.0	4.1	28.1	22.6	15.1	20.4	5.4

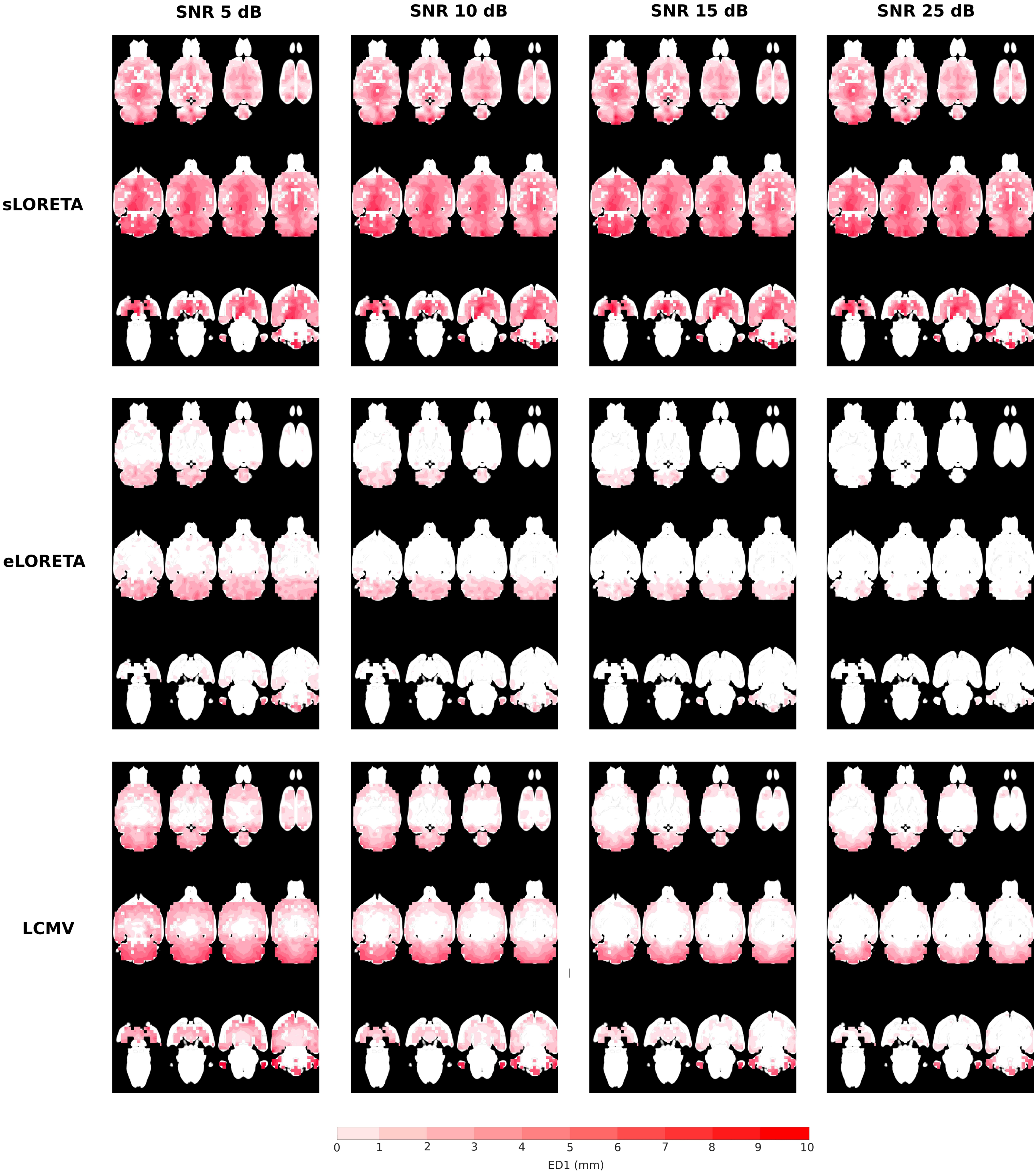

Figure S2. ED1 maps for sLORETA, eLORETA, and LCMV depending on a different SNR: 5, 10, 15, 25 dB

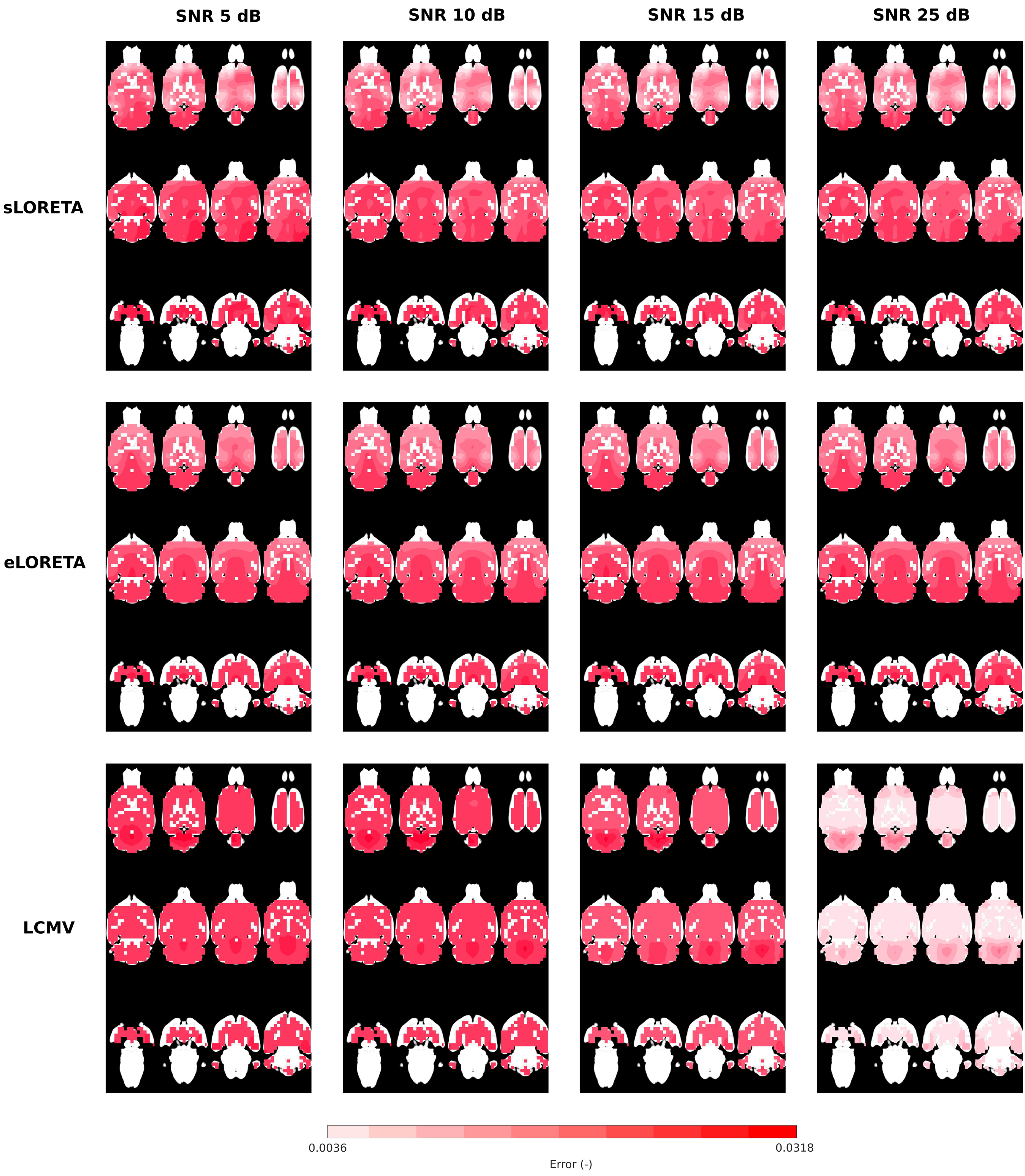

Figure S3. Reliability maps for sLORETA, eLORETA, and LCMV depending on a different SNR: 5, 10, 15, 25 dB

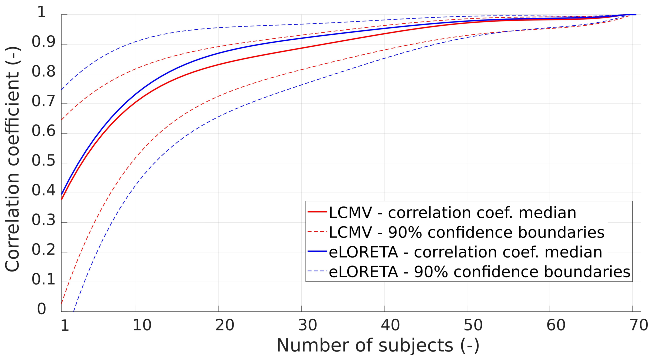

Figure S4. Median and 90% confidence boundaries of a spatial correlation coefficient of grand averaged estimated sources when a particular number of subjects (x-axis) was used for source localization, and grand averaged estimated sources when the whole dataset was used. For each point of the x-axis, a statistic was computed based on 70 different random combinations of subjects.
