# Supplementary figures and images for: Electrical Source Imaging in Freely Moving Rats: Evaluation of a 12-Electrode Cortical Electroencephalography System
Source: Front Neuroinform. 2021 Jan 25;14:589228. doi: 10.3389/fninf.2020.589228 (PMC7868391; doi:10.3389/fninf.2020.589228)

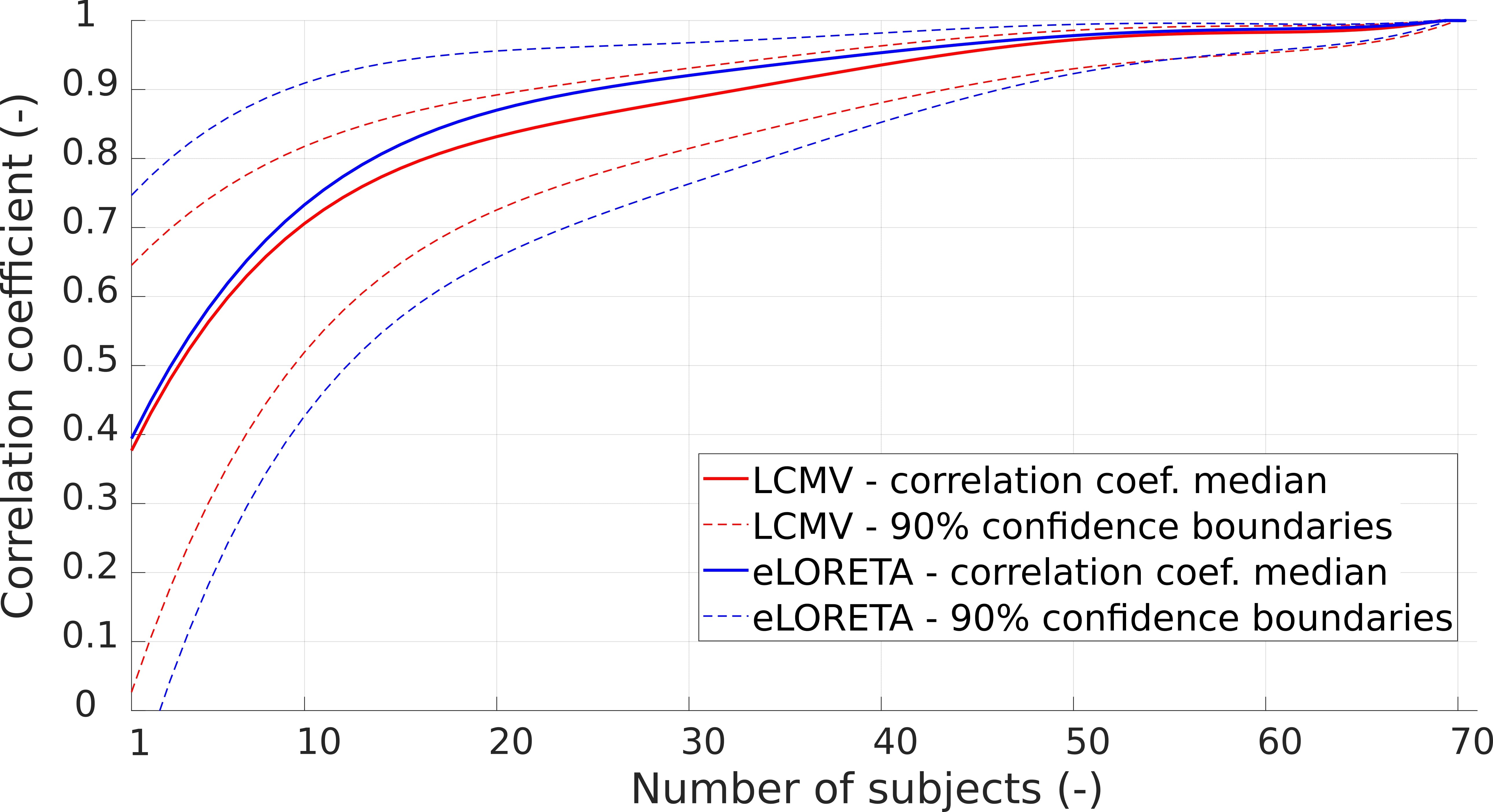

Supplement: Supplementary file 1 [file Data_Sheet_1.ZIP › data_size.jpg]

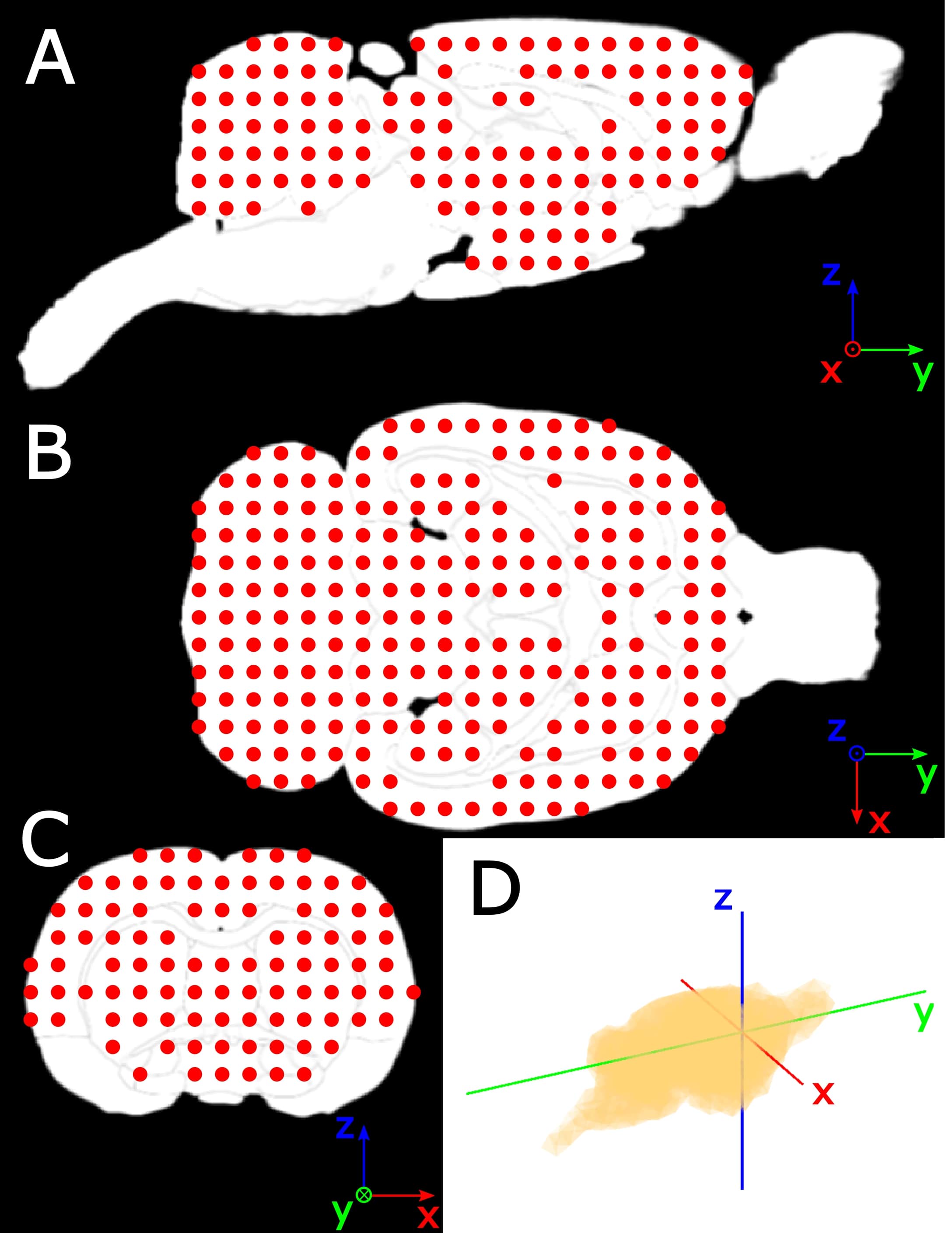

Supplement: Supplementary file 1 [file Data_Sheet_1.ZIP › sourcemodel.jpg]
